# Machine learning-supported identification of necrosis in colorectal and pancreatic cancer spheroids

**DOI:** 10.1371/journal.pone.0357972

**Published:** 2026-09-15

**Authors:** Miguel Martínez Lozano, Heinz D. Wanzenboeck, Sonia Prado-López

**Affiliations:** Institute of Solid State Electronics, Faculty of Electrical Engineering and Information Technology, TU Wien, Vienna, Austria; Nova Southeastern University, UNITED STATES OF AMERICA

## Abstract

**Significance:**

The transition to three-dimensional cell models is a reality in cancer research. While these models are more physiologically relevant, they present challenges that have not been previously considered. Certain aspects of 3D cultures, such as cell death, need to be modelled as they have a profound impact on spheroid survival and therefore on cancer treatment testing.

**Aim:**

To identify necrosis in HT-29 and Hs766-T cancer spheroids using an accurate, cost-effective, and fast machine learning-based system that relies only on brightfield (BF) images.

**Approach:**

In this study, three staining procedures with dyes were performed in colorectal and pancreatic cancer spheroids to highlight necrosis. The microscopic images of these stains serve as a ground truth for different machine learning models, which can subsequently learn to detect necrosis from the brightfield image, i.e., without the need for staining.

**Results:**

The results show a strong correlation between dark spots in the spheroid and their staining as necrotic, making regression models an appropriate solution. In general, the supervised models performed better than the unsupervised ones. The models perform worse in all cases for pancreatic Hs-766T spheroids, where necrosis is manifested as small, scattered spots, as opposed to colorectal HT-29 spheroids, where necrosis is a larger cluster in the center. The U-Net regression models demonstrated particularly noteworthy performance, at times surpassing 90% Normalized Coefficient of Correlation.

**Conclusions:**

The study findings demonstrate the potential for modeling necrosis in spheroids to a certain extent using 2D microscopy images and machine learning identification. The accuracy of the models largely depends on the manifestation of the necrosis, that is, the specific cancer cells that make up the spheroids.

## 1 Introduction

The modeling of tumoral cells and tissues has become a prominent area of research in recent years [1]. Cancer is a prevalent disease, with approximately 19.3 million new cases diagnosed in 2020. The annual number of cases is predicted to reach 28.4 million by 2040 [2]. In this context, colorectal cancer (CRC) is a salient example, accounting for 10% of all cancer cases and ranking as the second leading cause of death worldwide [2]. Conversely, pancreatic cancer (PC) is among the most aggressive diseases, presenting significant challenges in both detection and therapy. According to statistical data, pancreatic cancer accounted for more than 5% of cancer-related deaths in 2020, despite constituting a mere 2.5% of all cancer diagnoses [2]. This underscores the imperative necessity for novel approaches and technologies to facilitate early cancer detection and the development of more effective treatments. 3D biological tumoral models facilitate a better comprehension of tumor behavior, thereby enabling more precise drug screening tests [3–5]. However, certain aspects of 3D cultures, such as cell death, must be thoroughly modeled as they significantly influence the survival of spheroids and, consequently, the testing of chemotherapeutic and immunotherapeutic treatments.

**Fig 1:**
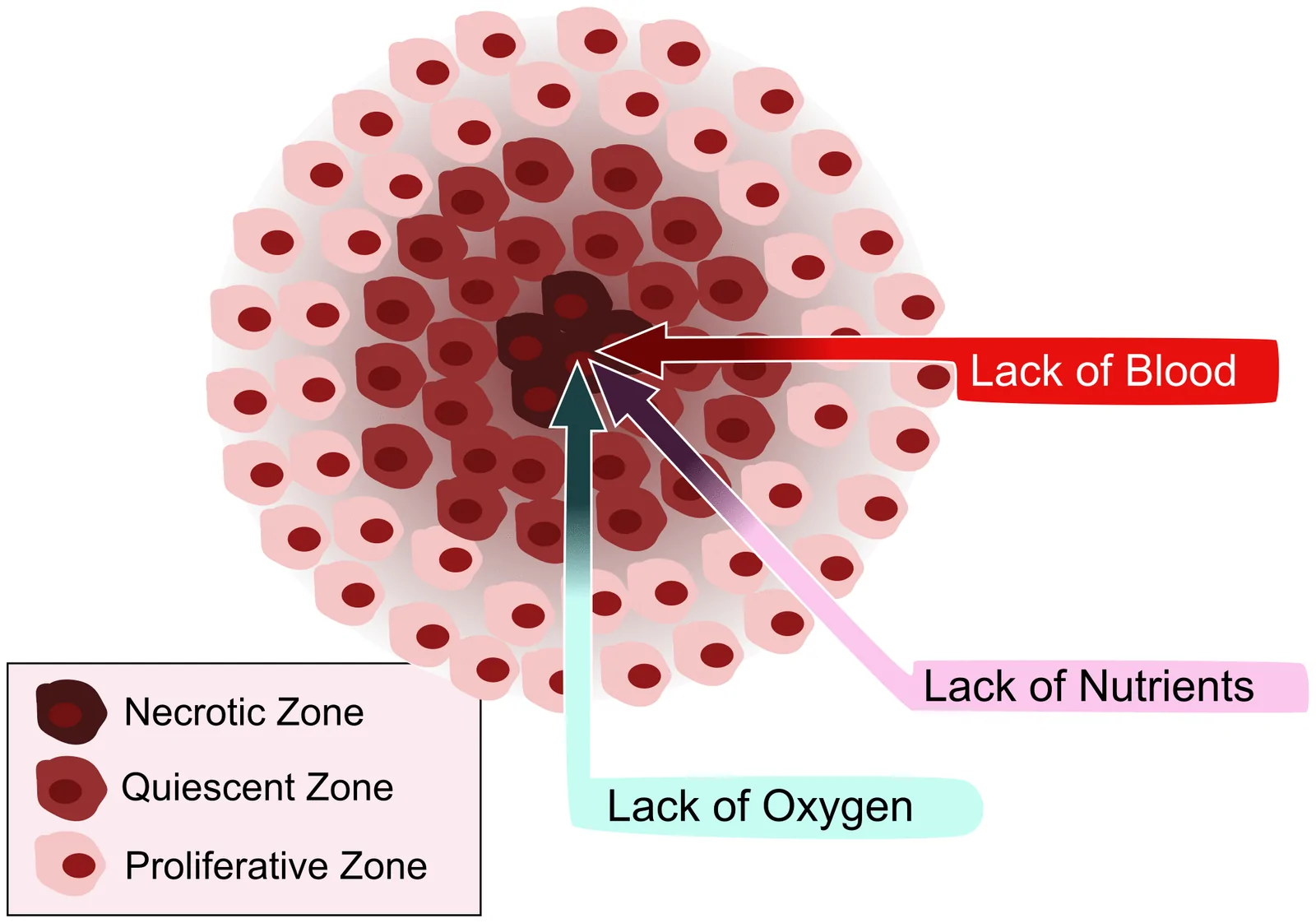
Modelling of Spheroid inherent Necrosis.

The present study focuses on the Computer-Vision aided detection of necrosis prior to *in vitro* drug screening and immunotherapy testing in CRC and PC tumor spheroids. Spheroids are a type of 3D cell culture tumor model [5]. They are self-contained and mimic cell-cell interactions in the human body better than traditional 2D tumor models [6]. Cancer treatment testing in 2D models has been shown to alter the response to treatments. A significant advantage of spheroids is that they do not necessitate genetic alteration for their formation, enabling the development of more reliable patient-derived models [7]. Furthermore, these models are capable of recapitulating oxygen gradients and chemoresistance behaviors observed in tumors [8]. Tumors in the body present an abnormal and inefficient vascular network [9]. Tumoral spheroids lack a vascular system. Although efforts are being made in this regard [3,10,11], the absence of vascularization leads to an uneven distribution of nutrients and oxygen and prevents effective metabolic waste removal. Consequently, the cells within the spheroid core undergo necrosis [3,12], as illustrated in Fig 1. It has been observed that a certain degree of necrosis occurs *naturally* in the spheroid, and this could affect the results of cancer therapy screenings on these tumoral models. The ability to accurately detect such necrosis will help to tackle this problem.

Computer Vision (CV) has proven effectivein similar contexts.; however, existing state is of the art focuses primarily on studying spheroid viability (extracted as a ratio from 0 to 1) [13,14], or extracting growth KPIs [15] from single Brightfield images using mostly Deep Learning Methods.

While these approaches offer valuable methodological insights, they address fundamentally different task formulations. Specifically, image to scalar regression. Our framework, nonetheless, requires image to image (or image to mask) translation models. Consequently, direct benchmarking against these pre-existing models was not methodologically feasible.

Resisting cell death is considered one of the *Hallmarks of Cancer*, which are known to contribute to the definition of this complex disease [16]. In certain situations, cancer cells evade apoptosis mechanisms [17]. This makes them resistant to agents that induce apoptosis to kill the tumoral cells [16,18]. The situation is different with other forms of cell death, such as autophagy and necrosis, as these processes can be both beneficial and harmful for the tumor. For instance, autophagy can lead to tumor regrowth after therapy [16], and necrosis can induce drug resistance or even tumor growth and angiogenesis [16,19]. An important process related to metastasis is necrosis, there is a large body of evidence that links the presence of necrosis inside solid tumors as colorectal cancer and pancreatic cancer as a strong indicator of a highly aggressive cancer and poor patient prognosis [20,21]. Dead cells cannot metastasize but alter the tumoral environment for the alive neighboring cancer cells to escape and spread throughout the body [22]. All previously mentioned underscore the critical importance of cell death control and monitoring in cancer therapy. Actual methods of detecting necrosis in multicellular tumoral spheroids, such as standard fluorescence live/dead staining followed by confocal microscopy [23,24], histological section followed by hematoxylin and eosin microscopic analysis [25,26], optical coherence tomography (OCT) [27], and Light sheet fluorescence microscopy to map exposed F-actin in necrotic cores [28], are often expensive, time consuming and destructive to the sample. To overcome these limitations, the present study explores possible Computer Vision systems for the automated detection of necrosis in cancer spheroids. The objective was to develop a cost-effective, fast, and precise automatic tool that segments necrosis in spheroids without the need for staining, relying exclusively on Brightfield (BF) images. To validate the efficacy of the proposed method in spheroids, its application was extended to two distinct types of cancer: colorectal and pancreatic cancer. To ensure the reliability of the results, adequate staining protocols were designed to establish a ground truth. These protocols facilitated the tracking of spheroids’ death over time, thereby enabling a more profound comprehension of their evolution.

## 2 Materials and methods

### 2.1. Cell expansion and Spheroid generation

In the present work, we used the CRC (HT-29) and the Pancreatic (Hs-766T) cell lines from ATCC. The cell lines were cultivated in 2D monolayers in accordance with the provider’s guidelines. The culture media for the CRC cell lines consisted of Dulbecco's Modified Eagle's Medium (DMEM (1X), Thermo Fisher Scientific Inc.), 10% v/v heat-inactivated Fetal Bovine Serum (FBS, Thermo Fisher Scientific Inc.), 1% v/v Gibco™ Penicillin Streptomycin (Pen Strep, Thermo Fisher Scientific Inc.) and 1% v/v Gibco™ L-glutamine 200 mM (Thermo Fisher Scientific Inc.). The cell culture medium was changed every two days. The cells were cultivated in an incubator maintained at 5% CO2 and at 37 ºC. The media was stored at 4 ºC and heated in a water bath to 37 ºC every time it was needed. To induce the formation of spheroids, the cells were cultivated in non-adherent 96-well plates (round-bottomed: Corning). Seeding densities 10,000 and 20,000 cells per well were utilized, with the cells being cultivated in media that was supplemented with 5 μg/mL insulin (GibcoTM), 10 ng/mL Fibroblast Growth Factor-Basic (bFGF, Merck KGaA), and 20 ng/mL of Epidermal Growth Factor (EGF, Sigma-Aldrich). It is noteworthy that the medium utilized for the spheroid cultures was not changed during the duration of the experiment.

### 2.2. Necrosis staining

For the detection of the necrotic area in the spheroids, we tested three dyes: 1) 4,6-Diamidin-2-phenilindol (DAPI), 2) Propidium Iodide (PI), and 3) Triphenyl-Tetrazolium Chloride (TTC). The first two are fluorescent, whereas TTC has been used to dye necrosis on the macroscopic scale [29]. DAPI is a dye that can bind to different DNA sequences. It is cell permeable at high concentrations, thus enabling its use for the fluorescent staining of both live and dead cells [30,31]. Its absorption peak is at ~350 nm, and its emission peak is at ~450 nm [30]. PI has been widely used to detect necrotic and apoptotic cells in combination with Annexin V^32^. The occurrence of apoptosis and necrosis in cells is accompanied by the manifestation of damaged cellular membranes. This allows PI to penetrate the cells and intercalate in the DNA of damaged and dead cells. Excitation of PI by green light (peak at 488 nm) results in the emission of red light (peak at 605 nm) [32,33]. TTC is a widely utilized stain for detecting damage or necrosis in structures such as organs, particularly in the context of stroke [34,35]. The underlying principle is rather simple: TTC is *uncolored*, but it can be reduced by the mitochondria into formazan, a red-*colored* compound that is insoluble in water and non-toxic in low concentrations, staining all tissues that carry cellular respiration [34–36]. In summary, all living tissues will exhibit a red staining due to the presence of TTC.

The TTC assay was performed on 24 HT-29 spheroids seeded in round-bottomed plates. The TTC assay was evaluated at six different concentrations (0.07, 0.3, 0.6, 1.2, 1.7 and 2 mg/mL). Four replicates were performed. In addition to the visual assessment, the assay was quantitatively evaluated by measuring the absorbance of the samples with an Enzo Absorbance 96 Plate Reader (Enzo Life Sciences, Inc., USA) at a wavelength of 450 nm.

Spheroids from colorectal cancer and pancreatic cancer were generated in round-bottomed plates as previously. Seeding densities 10,000 and 20,000 cells per well were utilized (four replicates per condition and exposure time). We allowed the formation of spheroids for 7 days. Afterwards, the spheroids were followed and studied for necrosis formation over two weeks. Data collection timelines were tailored to the specific growth kinetics and structural stability of each cell line. HT-29 colorectal cancer spheroids remained viable and structurally intact until day 18, whereas Hs-766T pancreatic cancer spheroids underwent more rapidly towards structural degradation, limiting reliable analysis after days 7–14. To maximize data yield from the more lasting HT-29 model, both cell lines were monitored through their maximum viable lifespans rather than standardizing to a shortened, uniform endpoint.

To mitigate potential toxic side effects associated with the dyes, staining was performed daily in situ. The concentrations of DAPI and PI utilized in this study were 62.5 µg/mL and 32.36 µg/mL, respectively. Imaging was performed using a microscope-embedded camera (Hamamatsu ORCA Flash 4.0). For each spheroid, a Brightfield (BF), a DAPI, and a PI image were obtained.

### 2.3. Image processing

There are no technical replicates in this study, i.e., all the images are from biological replicates. We processed the images using *Python*. All the codes are available in GitHub (https://github.com/hibiscus22/NecrosisDetector/), and all models can be trained in the free version of Google Colab Notebooks, also added in the repository. We tested several computer vision programs, including:

#### Otsu’s algorithm.

This algorithm converts a grayscale image into a two-color image (i.e., binary mask) using the pixel-value threshold that ensures maximum variance [37]. It is very fast and shows efficacy in many contexts. We used Otsu’s algorithm to 1) Automatically Generate Masks from DAPI and PI Images, 2) Delineate the Brightfield spheroid image to match DAPIs output.

#### Watershed algorithm.

It is a classic segmentation algorithm, which can be considered a good benchmark for most computer vision applications of this kind. It is important to note that this algorithm necessitates a certain degree of pre-processing to identify specific features of the object [38].

#### Logistic regression.

Machine-learning technique that has been developed for analyzing binary data. This algorithm is supervised, thus necessitating a training dataset. The trained model predicts the probability of a new value being 1 [39].

#### Decision tree classifier and regressor.

It is a machine learning algorithm that employs a tree-based structure for data visualization and the decision-making process. This is trained to generate a series of straightforward decisions that categorize or predict a probability [40].

#### U-Net.

The only Deep Learning algorithm tested. It consists of two Convolutional Neural Networks in a symmetric architecture, such that the first one works as an encoder and the second one as a decoder. The implementation of the network was conducted without same-dimensional space feedback [41].

#### Watershed after K-Means (K = 3) (referred to as WaterMeans).

K-Means is an unsupervised machine-learning clustering algorithm that divides any group of data into the indicated number of clusters. In this work, *K* was set to 3 to divide the spheroid into Necrotic, Quiescent, and Proliferative zones. We generate an image in which every pixel is a binary *mask* (Fig 4). The Watershed algorithm attempts to expand from this point in all directions until it finds a border [42]. For classical Machine Learning algorithms (Logistic Regression and Decision Trees), a 70/30 train/test split was applied. The exact number of spheroid replicates corresponding to these splits is detailed in Table 1. For Deep Learning architectures, a 5-fold cross-validation scheme was implemented to mitigate overfitting and ensure robust performance evaluation.”

**Table 1:** The following data table presents the number of CRC and PC spheroids that were imaged, categorized according to cell line, cell doses, and age in days. Two different cell doses were tested: 10,000 cells and 20,000 cells.

Cell line/Age (days)	7	8	9	10	12	14	15	16	17	18	ML Train/Test Split	Total
HT-29(10k)	4	4	4	4	4	4	3	2	3	0	49/21	70
HT-29(20k)	4	4	4	4	4	4	4	3	4	3		
Hs-766T(10k)	4	4	4	4	–	–	–	–	–	–	22/9	30
Hs-766T(20k)	4	4	4	3	–	–	–	–	–	–		
Total	–	–	–	–	–	–	–	–	–	–	71/30	101

There are two main ways to analyze the images. The first approach involves the classification of each pixel in the *brightfield* microscopy image of the spheroids as white or black, i.e., dead or alive.

Using Masks is a simplistic approximation, as there are hundreds of cells in each pixel, and the staining is brighter in the more necrotic areas. The second approach involves direct prediction of the Stain Image from the Brightfield Image.

### Machine learning

**Fig 2:**
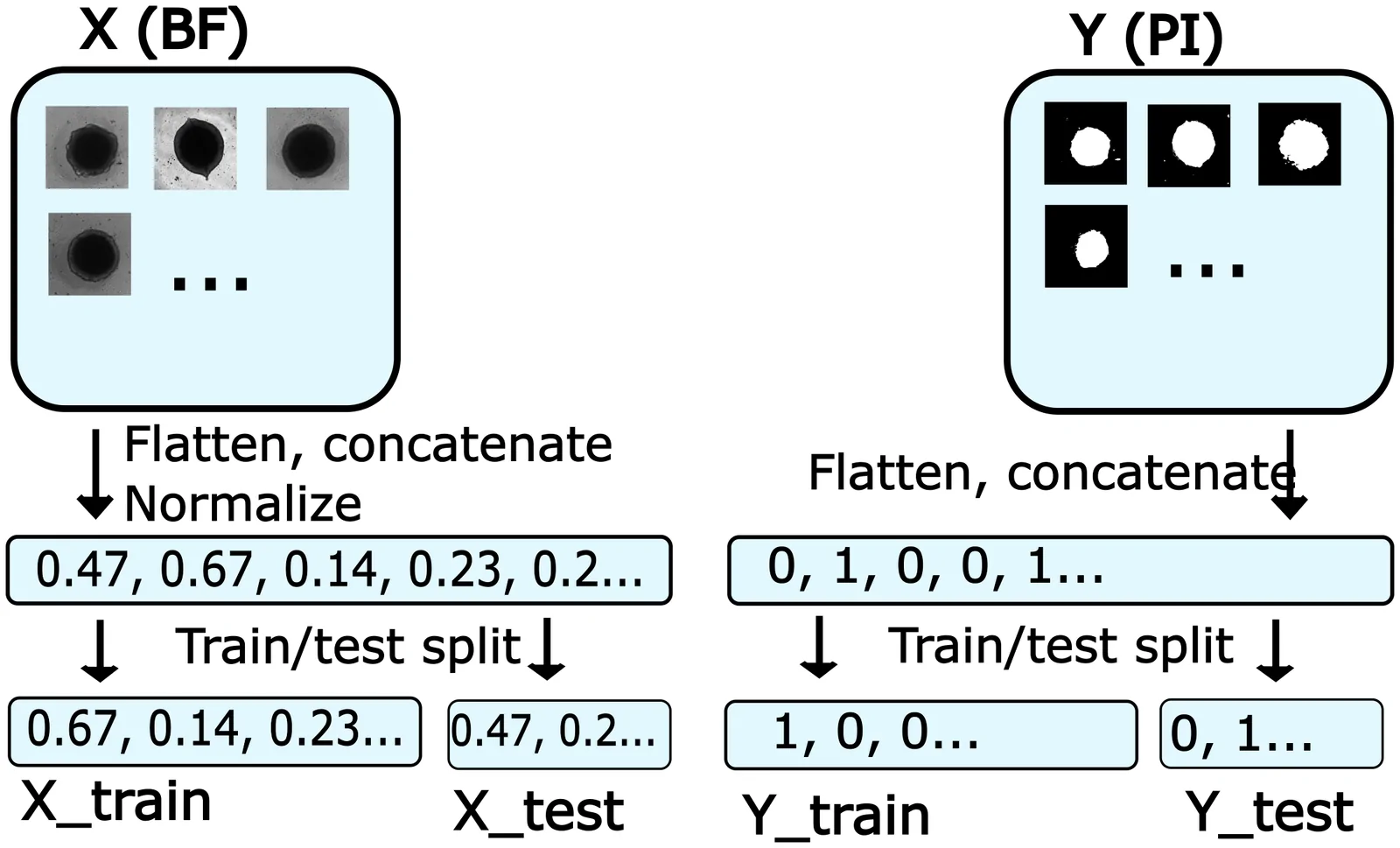
Machine Learning data workflow. The intensity of the pixel is normalized to correlate pixel intensity with necrosis.

Machine learning (ML) methods were designed and trained *ad hoc* to demonstrate a fundamental principle and prove a basic idea: there is a correlation between the dark intensity of the core zone of the spheroids (Brightfield images) and the staining intensity of the necrotic zone (Stained images). The staining intensity of the necrotic zone is contingent on how dark the core of the Brightfield spheroid appears. Consequently, the sole variable integrated into the machine learning models pertained to pixel intensity (see Fig 2). This methodological approach facilitated the training of both classification and regression models. For these algorithms, the dataset was split into Train and Test groups, 70/30. The following parameters were used:

1. *Logistic Regression – Scikit Learn: C = 1.0, l1_ratio = 0.0, dual = False, tol = 0.0001, fit_intercept = True, intercept_scaling = 1, class_weight = None, random_state = None, solver = ’lbfgs,’ max_iter = 1000*
2. *Decision Tree Classifier – Scikit Learn: criterion = ’gini,’ splitter = ’best,’ max_depth = None, min_samples_split = 2, min_samples_leaf = 1, min_weight_fraction_leaf = 0.0, max_features = None, random_state = None, max_leaf_nodes = None, min_impurity_decrease = 0.0, class_weight = None, ccp_alpha = 0.0, monotonic_cst = None*
3. *Decision Tree Regressor – Scikit Learn: criterion = ’squared_error,’ splitter = ’best,’ max_depth = None, min_samples_split = 2, min_samples_leaf = 1, min_weight_fraction_leaf = 0.0, max_features = None, random_state = None, max_leaf_nodes = None, min_impurity_decrease = 0.0, ccp_alpha = 0.0, monotonic_cst = None*

**Fig 3:**
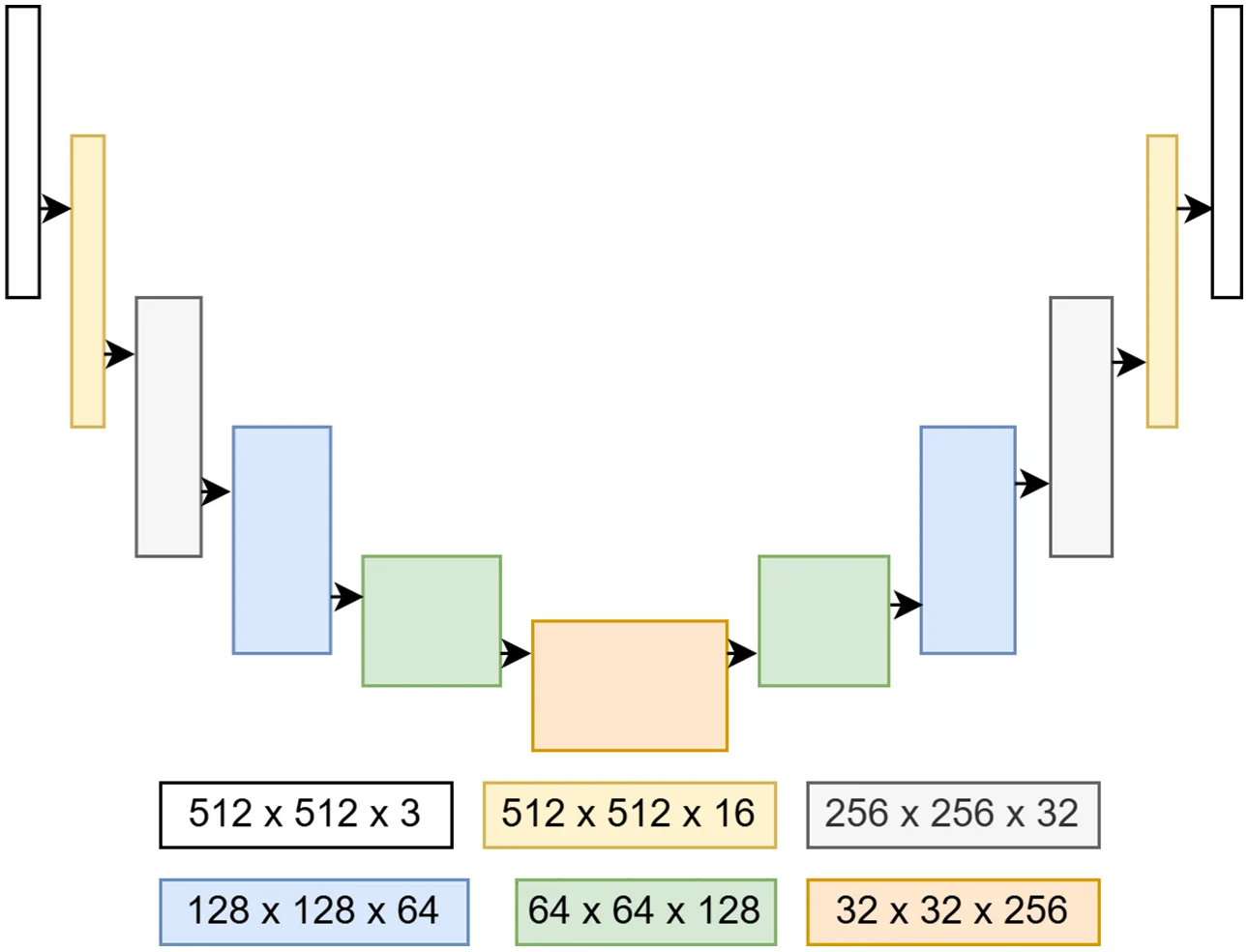
UNet architecture schematic.

As for deep learning (DL), existing segmentation-oriented architectures were adapted to address the specific problem at hand. We opted for a U-Net model [41]. A validation group was **not** created due to the small dataset size. To avoid overfitting, we did a 5-Fold Cross Validation. The UNet we implemented has a 5-layer architecture, as shown in Fig 3. Each layer comprises:

1. ReLU-activated, same padding Convolution.
2. Dropout of 0.1 in upper levels, 0.2 in medium, 0.3 in lower levels, to favor smaller features.
3. ReLU-activated, same padding Convolution.
4. Average (binary) or Max (regression) Pooling Layer.

And was trained using these hyperparameters:

- Optimizer: Adam, Learning Rate 1e-5
- Epochs: 70
- Batch size: 1
- Loss function: Binary Cross Entropy
- Metric: Binary Accuracy (binary), Mean Squared Error (regression)

### Solution metric

**Fig 4:**
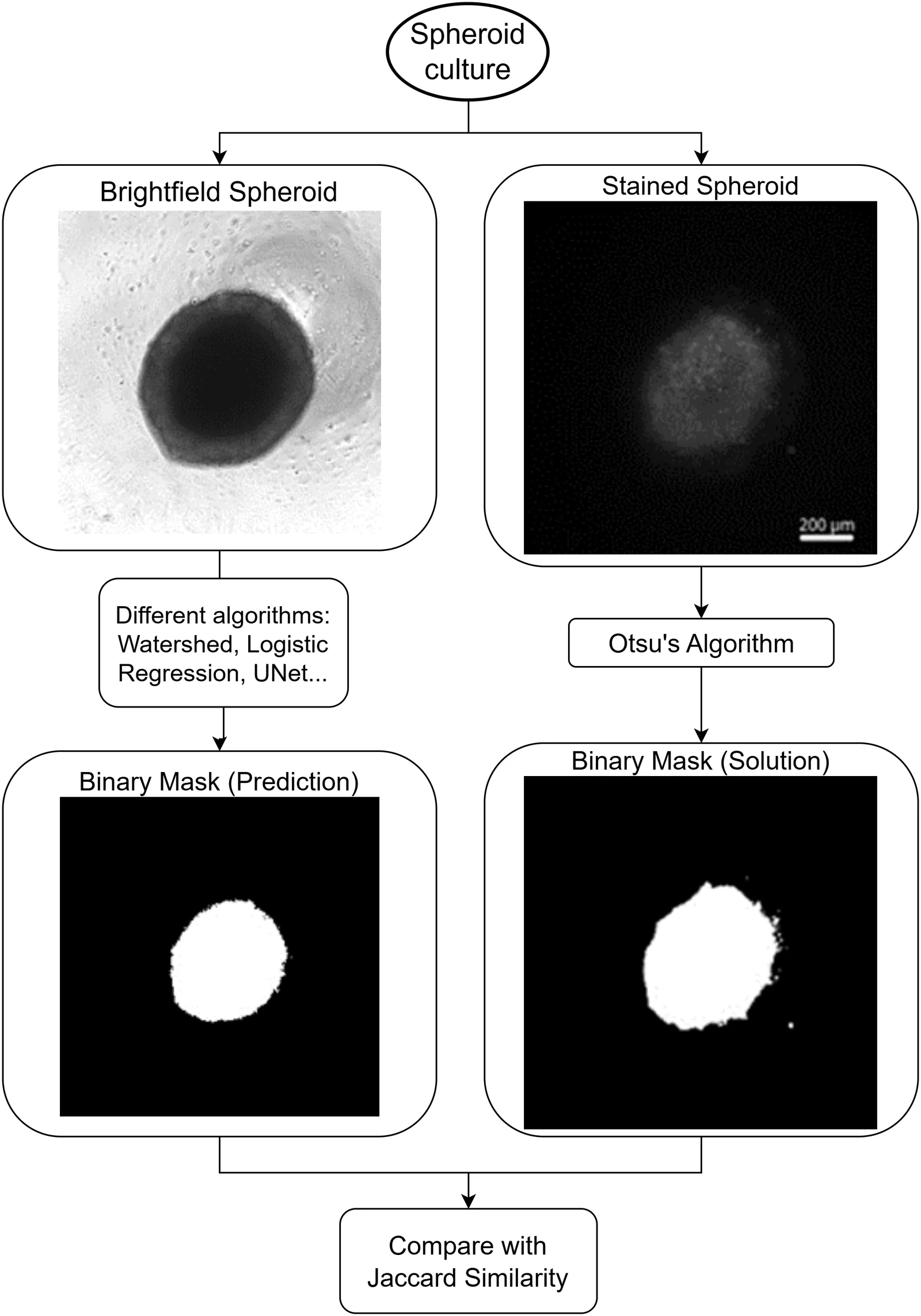
Flow diagram of image generation and comparison.

Binary masks are automatically created from the *Stained Image* using Otsu’s algorithm simple technique. Why? We need a uniform, impartial method to decide which pixels are going to be 0 and which are going to be 1. A simple threshold could work perfectly, but Otsu’s method maximizes the variance between the 0 and 1 classes, thus creating masks of higher quality that better represent the staining. We generate an image in which every pixel is a binary *mask* (Fig 4):

Afterwards, we generated Prediction Solution masks from Brightfield Images with the above-mentioned Computer Vision Techniques. The need for a quantitative metric of the similarity between Prediction (P) and Solution (S) led to the implementation of *Jaccard Similarity* (J) [43], shown in the (Equation 1).


$$\begin{array}{c} J=\frac{P\cap S}{P\cup S} \end{array}$$ (1)

Equation 1 Jaccard Similarity (J) between Prediction (P) and Solution (S) binary sets. The range of this value is from 0 to 1.

Jaccard Similarity is ideal for binary data, as it is the ratio between the pixels the Prediction system got right and the total number of positive pixels. It is easy to implement and grants good intuition (Jaccard Similarity of 50% means the system got half of the necrotic pixels right) [43].

The second approach entails a comparison of whole images to obtain a percentage of similarity. The *Normalized Coefficient of Correlation (NCC)* was selected as the metric for evaluating the similarity between images. This coefficient, based on convolving one image over the other, ranges from −1–1 is fast, simple, and well-proven and ubiquitous in signal and image analysis [44]. Additionally, it is quite intuitive to understand: two identical images have an NCC of 1, one image and its negative have a −1 NCC, and one image with random noise will have an NCC very close to 0. NCC has a flaw: it is not robust against rotations. In this study, we do not compare rotated spheroids; thus, this method is consistent.

## 3 Results

### 3.1. Spheroid structure and necrosis distribution in the stained images

**Fig 5:**
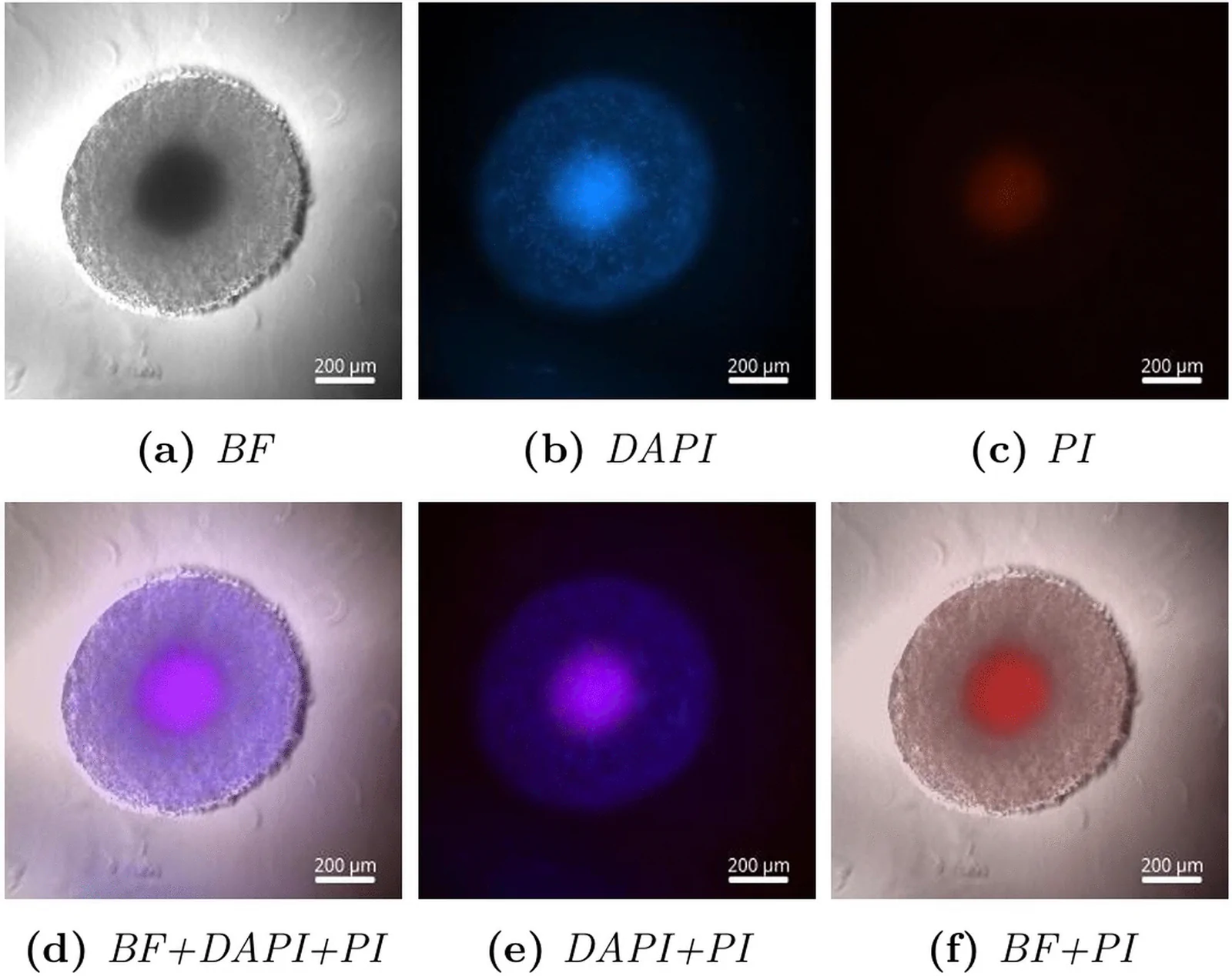
Colorectal cancer 8-day-old spheroids from HT-29. Brightfield, DAPI, and PI staining.

The utilization of DAPI and PI enables the acquisition of fluorescence images of the proliferative/quiescent and necrotic areas of the spheroids, as evidenced in Fig 5.

In this study, a total of 101 spheroids were grown over the course of several days and stained as shown in Table 1.

**Fig 6:**
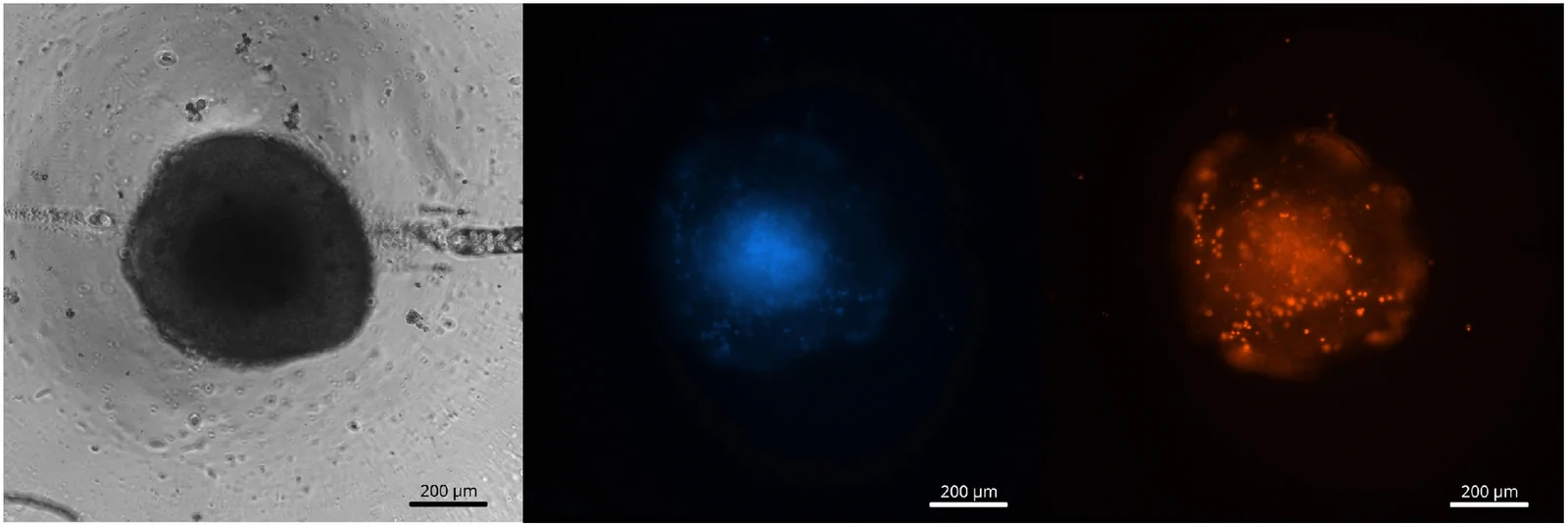
Colorectal cancer 8-day-old spheroids from HT-29, Left: Brightfield, Centre: DAPI, Right: PI staining.

The images of HT-29 exhibited the following characteristics, as illustrated in Fig 6.

**Fig 7:**
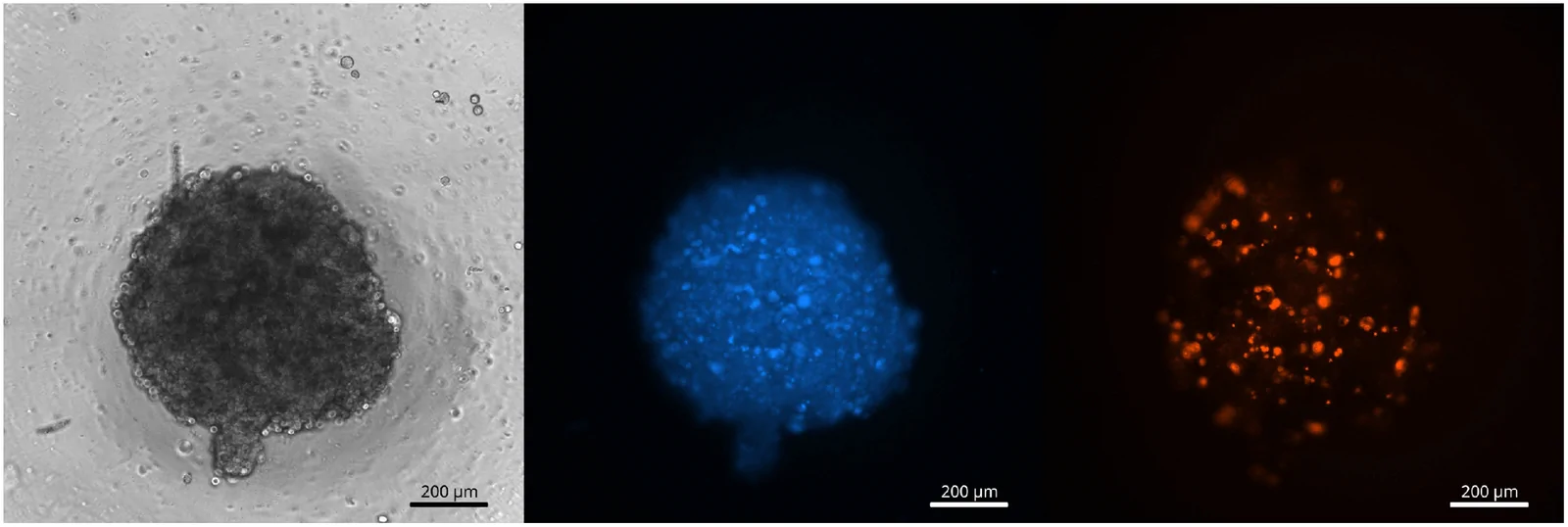
Pancreatic cancer 7-day-old spheroids from Hs-766T. Left: Brightfield, Centre: DAPI, and Right: PI staining.

Whereas Pancreatic Hs-766T spheroids exhibited the following characteristics (Fig 7):

A total of three images were obtained for each spheroid, encompassing BF, DAPI, and PI channels.

### 3.2. Necrosis prediction using computer vision

**Fig 8:**
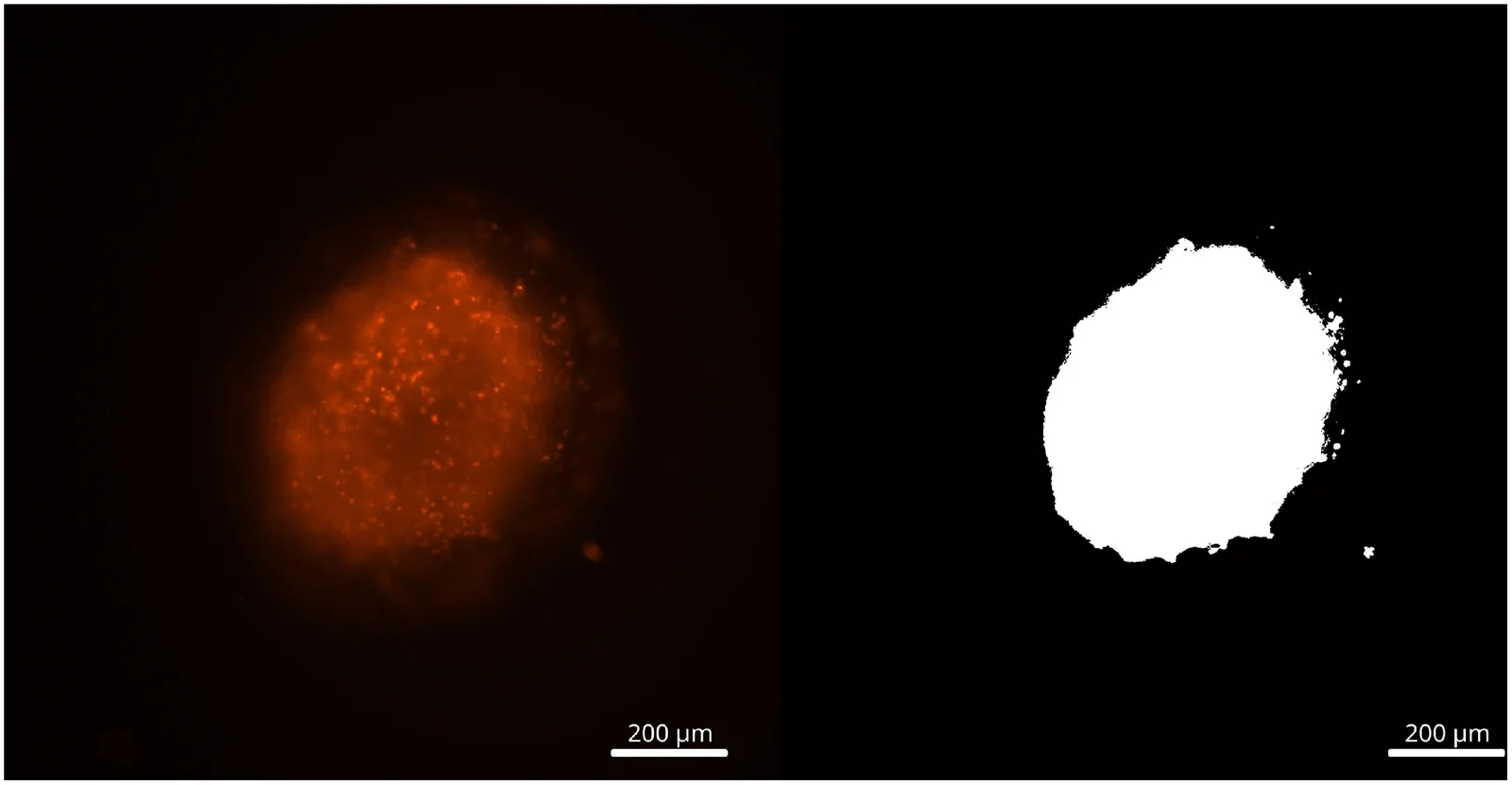
Binary mask of a PI fluorescence staining image. This was created by applying Otsu’s algorithm [37] directly to the fluorescence image. This procedure is entirely automatic and based only on the threshold that maximizes variance between zones.

After this, binary masks were generated for all images employing Otsu's algorithm (see Fig 8).

**Fig 9:**
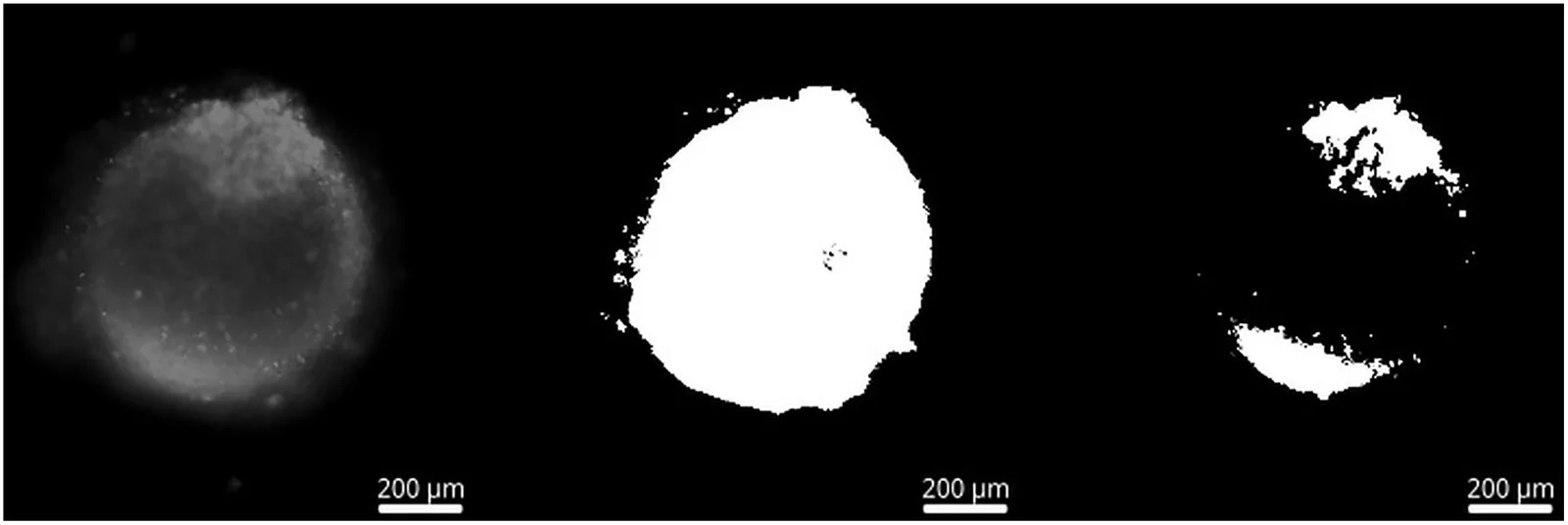
Dichotomy on spheroid mask creation. Two equally valid solutions are displayed.

The segmentation can be done in a different fashion (Fig 9), as will be detailed in the Discussion.

**Fig 10:**
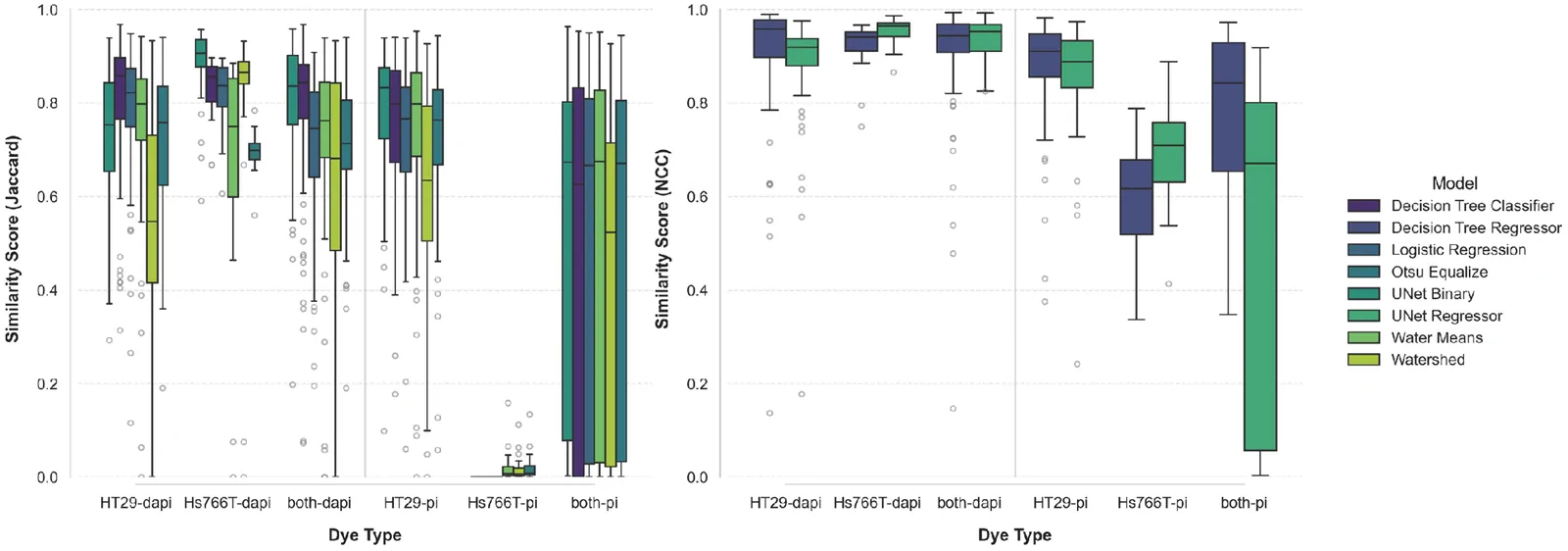
Model performance breakdown. Left: Mask-based models. One-way ANOVA was carried out to assess significant inter-model (p < 0.01) and inter-dye (p < 0.01) differences. Right: Regression-based models. T-Test was carried out to assess significant inter-model (p < 0.01) and inter-dye (p < 0.01) differences.

To achieve the most accurate result possible, several algorithms were implemented on the BF images. Subsequently, a comparison was made between the two masks. After this, a procedure was executed in which image-to-image models were trained Fig 10. The ensuing results are displayed in Table 2:

**Table 2:** Top: Success rate overview of all models tested. Discrete Masks: Jaccard Similarity. Continuous solution: NC Coeff. Bottom: ANOVA summary results. Display format: Mean [95% confidence interval].

Model	HT29-dapi	HT29-pi	Hs766T-dapi	Hs766T-pi	both-dapi	both-pi
Decision Tree Classifier	0.799 [0.756, 0.830]	0.746 [0.699, 0.780]	0.833 [0.807, 0.850]	0.0 [0.0, 0.0]	0.783 [0.743, 0.813]	0.468 [0.388, 0.541]
Logistic Regression	0.778 [0.732, 0.810]	0.725 [0.680, 0.759]	0.822 [0.794, 0.842]	0.0 [0.0, 0.0]	0.711 [0.677, 0.738]	0.484 [0.413, 0.554]
Otsu Equalize	0.717 [0.676, 0.751]	0.719 [0.668, 0.754]	0.696 [0.681, 0.708]	0.019 [0.012, 0.032]	0.71 [0.681, 0.734]	0.498 [0.424, 0.565]
UNet Binary	0.730 [0.693, 0.762]	0.780 [0.737, 0.809]	0.880 [0.842, 0.902]	0.0 [0.0, 0.0]	0.800 [0.770, 0.823]	0.513 [0.436, 0.586]
WaterMeans	0.747 [0.694, 0.782]	0.734 [0.676, 0.778]	0.686 [0.593, 0.747]	0.019 [0.012, 0.037]	0.72 [0.674, 0.751]	0.502 [0.425, 0.573]
Watershed	0.562 [0.517, 0.608]	0.626 [0.575, 0.672]	0.806 [0.679, 0.857]	0.016 [0.010, 0.027]	0.639 [0.592, 0.682]	0.43 [0.364, 0.497]
Decision Tree Regressor	0.901 [0.854, 0.928]	0.873 [0.839, 0.898]	0.924 [0.901, 0.936]	0.598 [0.558, 0.633]	0.911 [0.880, 0.929]	0.78 [0.745, 0.812]
UNet Regressor	0.882 [0.841, 0.903]	0.861 [0.825, 0.883]	0.954 [0.943, 0.962]	0.694 [0.657, 0.725]	0.936 [0.920, 0.949]	0.504 [0.433, 0.570]

### ANOVA

**Table:** 

Source	pvalue(binary)	pvalue(continuous)
Model	4.09E-13	1.13E-06
Dye / group	3.51E-306	3.32E-61
Model*dye	4.73E-04	1.21E-19

The Watershed algorithm can be considered as the classical CV benchmark to compare against.

### 3.3. Prediction in CRC spheroids

**Fig 11:**
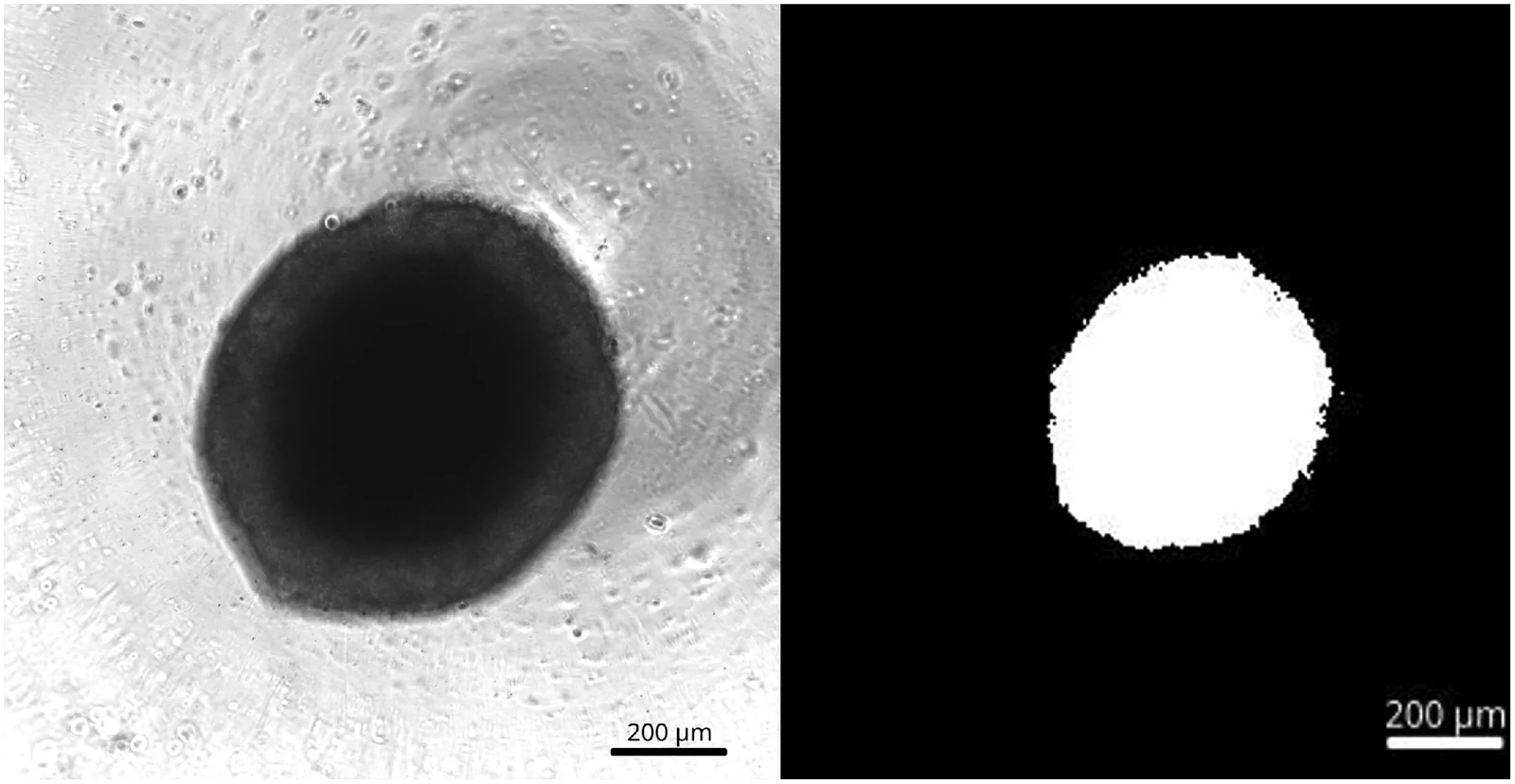
Extraction of a necrosis map of an HT-29 spheroid. On the left side of the figure, a BF image of the spheroid is presented. The right image depicts: Prediction made by our Decision Tree Classifier.

**Fig 12:**
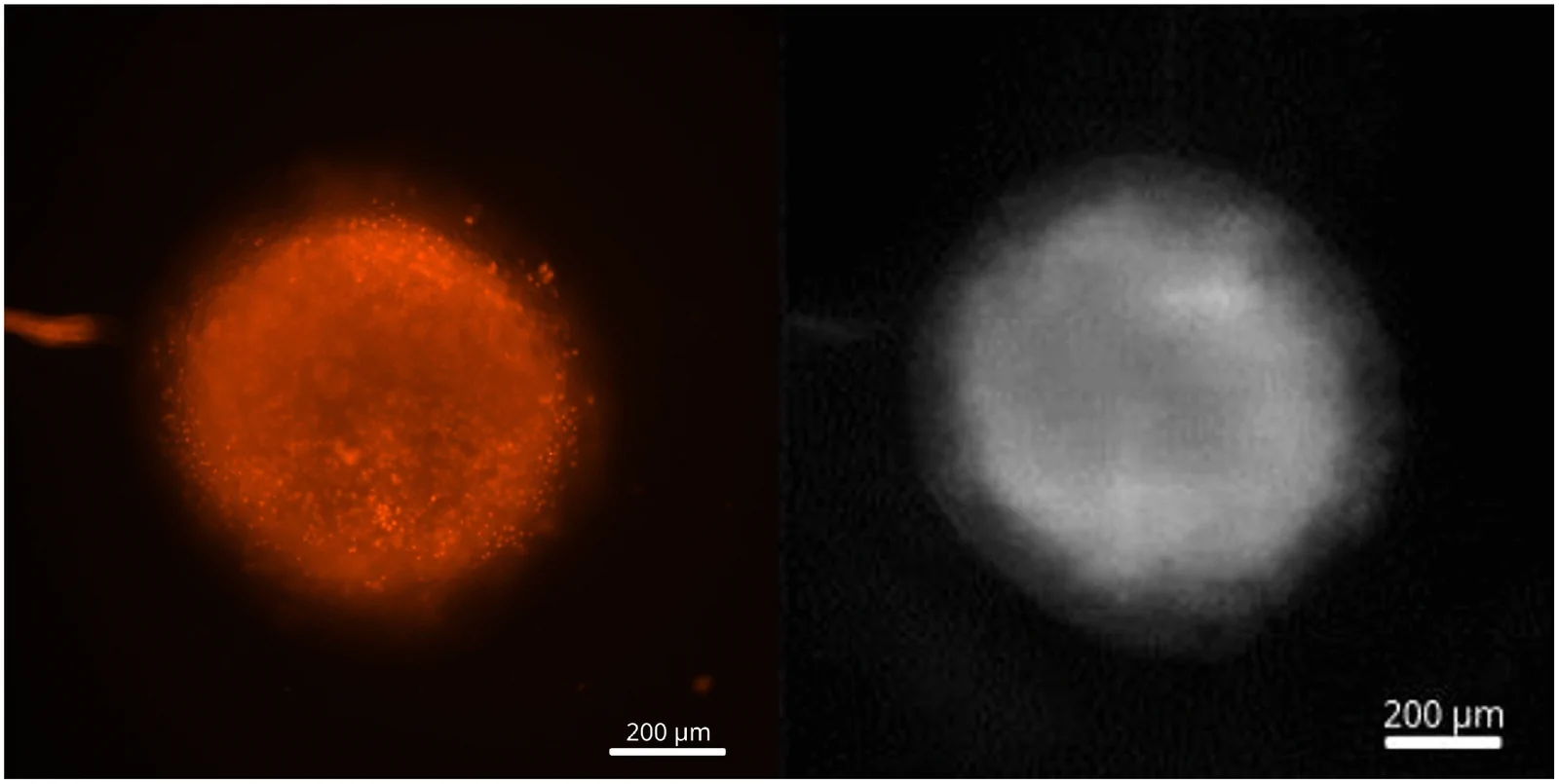
On the left side of the figure, PI staining of an HT-29 spheroid is shown. Right: Prediction of a U-Net of the image on the left. NCCoeff: 0.95.

This dataset contains images of spheroids taken over one week. We predicted necrosis using, among others, ML (Fig 11) and DL (Fig 12) techniques.

### 3.4. Prediction in PC Spheroids

**Fig 13:**
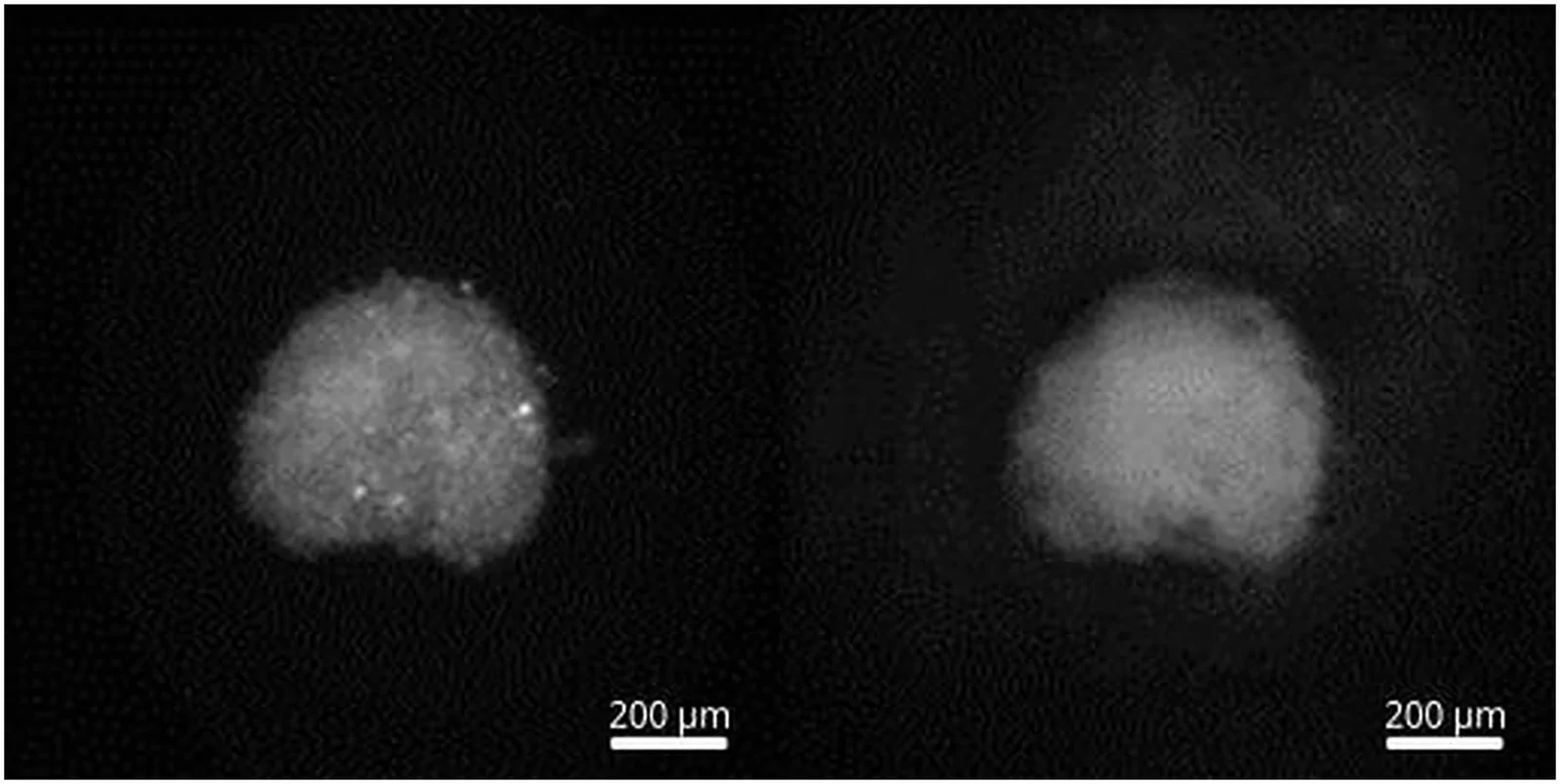
Output of UNet (right) for a DAPI staining of an Hs766-T spheroid (left). NCCoef: 0.95.

**Fig 14:**
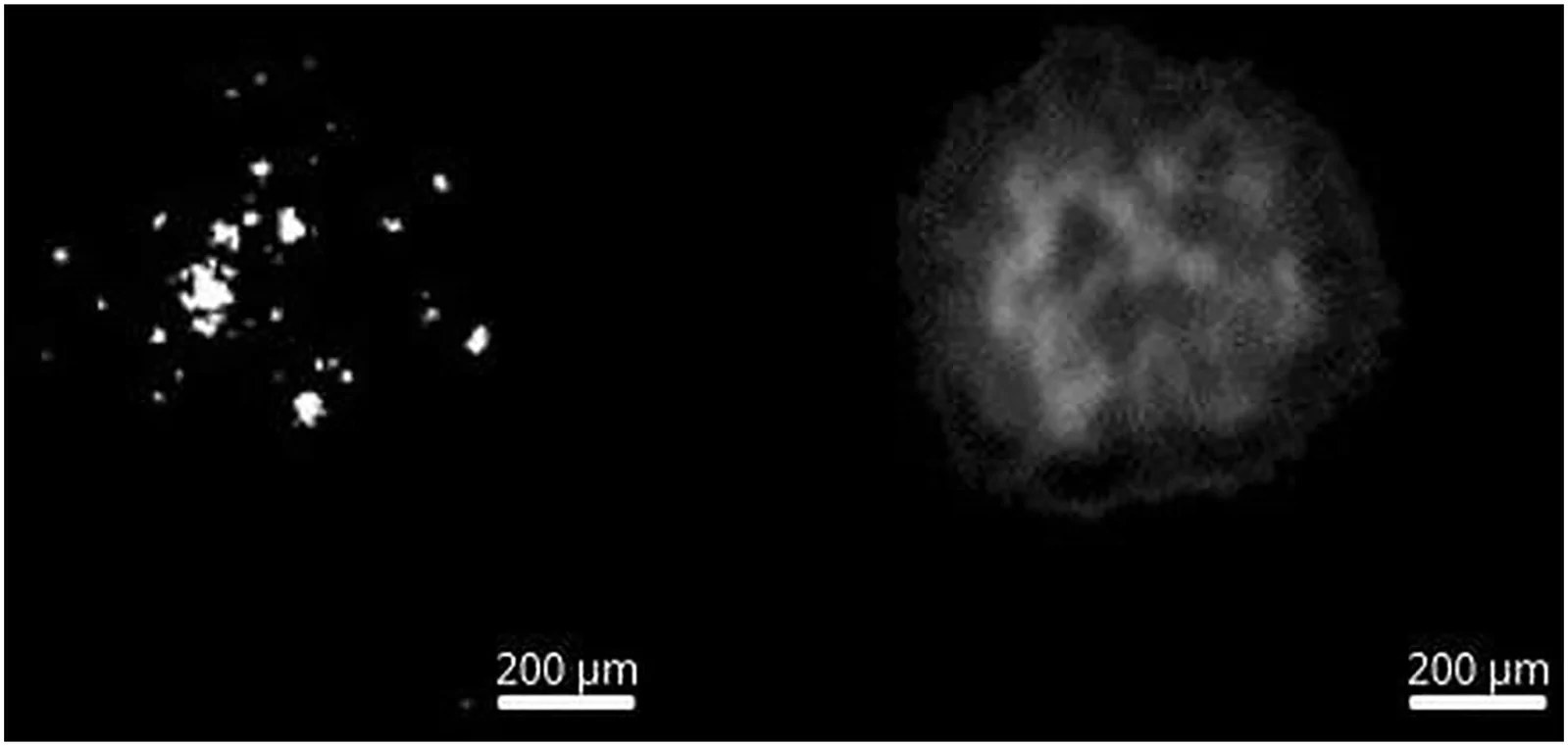
Output of U-Net (right) for a PI staining of a Pancreas spheroid (left), trained with images of both spheroids. NCCoef: 0.6.

This dataset is smaller; thus, the resulting models are more limited. DL models are shown for DAPI results (Fig 13) and PI (Fig 14).

**Fig 15:**
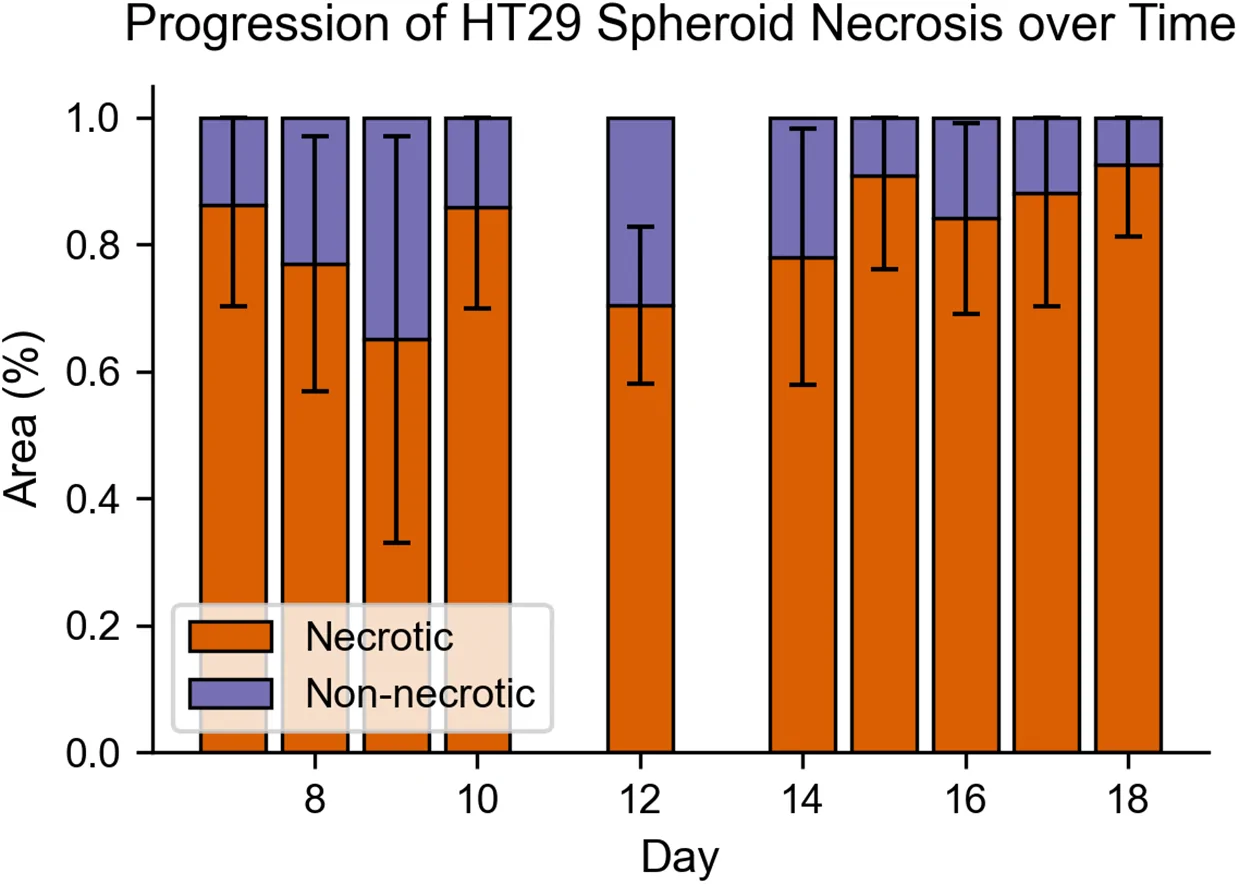
Distribution of the dead and alive areas in HT-29 Colorectal Cancer Spheroids over time. The dead areas (red) are compared to the non-dead areas (blue).

**Fig 16:**
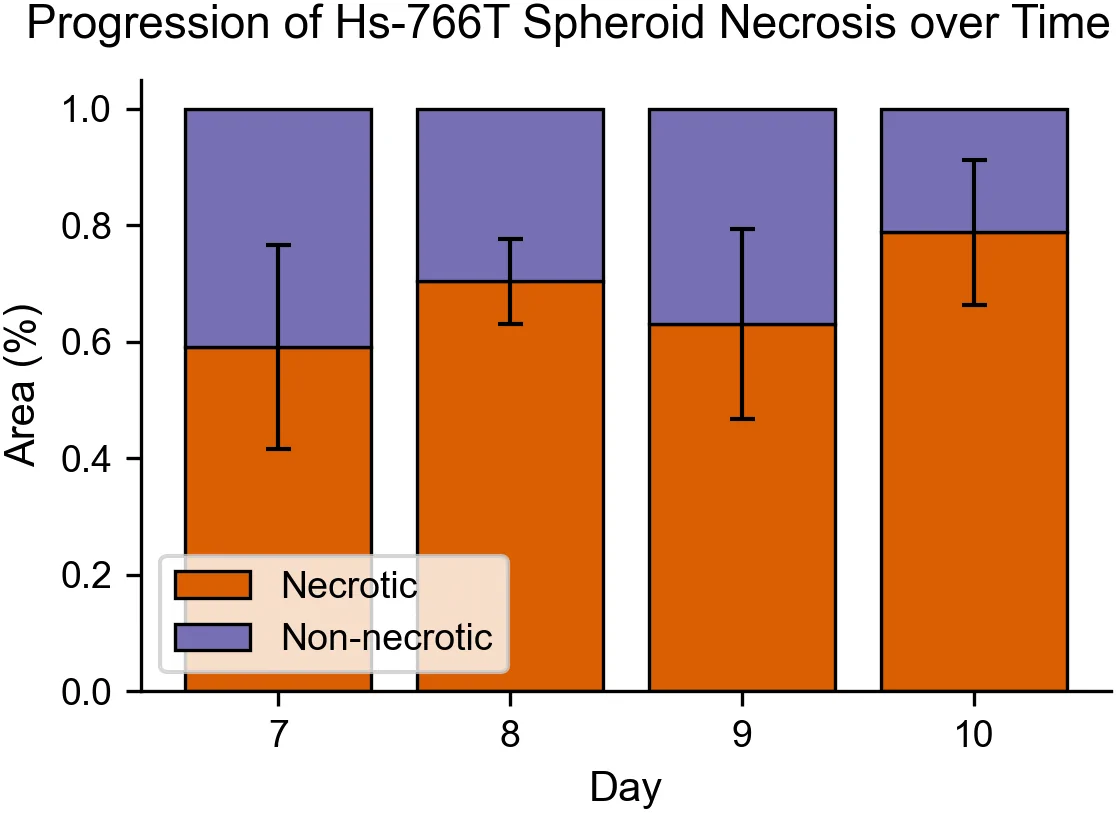
Distribution of the dead and alive areas in Hs-766T Pancreatic Cancer Spheroids over time. The dead areas (red) are compared to the non-dead areas (blue).

We also monitored the viability of the spheroids, distinguishing between viable and non-viable regions. To this end, the DAPI-stained number of pixels is determined to find the total spheroid area, while PI pixels allow identifying the dead area. After this, the difference between PI and DAPI is calculated, yielding the area of the living part of the spheroids. The distribution of the alive and dead areas over time is illustrated in Fig 15 and Fig 16, respectively.

## 4 Discussion

### 4.1. Spheroid’s structure and necrosis distribution in the stained images

The stained images with DAPI and PI in pancreatic Hs-766T and colorectal HT-29 spheroids exhibited a clear definition. As illustrated in Fig 5, a standard example of the problem to be addressed, the spheroid shows a necrotic core, as indicated by PI, and a proliferation/quiescent zone stained by DAPI. CRC spheroids exhibit increased necrotic characteristics in the round-bottomed plate compared to the flat-bottomed one (Fig 5). This observation can be attributed to the fact that CRC spheroids have a comparatively smaller surface area in contact with the media (due to geometry) and a larger size overall, resulting in a deprivation of oxygen and nutrients from the media. In contrast, pancreatic spheroids are less impacted by necrosis. Our analysis revealed that necrosis was not concentrated at the core, as observed in CRC spheroids (Fig 7). Instead, necrosis manifests in small clusters distributed across the entire structure. The core is still more necrotic than the periphery, predicting where the cluster will appear is nonetheless unattainable to our models. This phenomenon can be attributed to the anatomical structure of the pancreas endocrine gland and the patches of endocrine tissue located within them (islets of Langerhans) [45]. Also, it has been shown that the formation of pancreas spheroids in concave microwells reduces the necrosis area size [46].

### 4.2. Limitations

Besides the above-mentioned pancreatic spheroid challenge, this study is marked by two main constraints: the size of the dataset and the extrapolation of three-dimensional features from two-dimensional images. Other limitations include the number of dyes (a second necrosis stain like indocyanine green can provide a second source of data), and the inability to differentiate whether the dead cells are apoptotic.

We examine three primary types of algorithms, each of which exhibits notable efficacy in specific contexts.

1) ***Traditional Computer Vision Algorithms***. They are characterized by their cost-effectiveness, speed, adaptability, and reliability. They are unsupervised and not subject to the constraints imposed by supervised learning. However, they exhibit limited flexibility and generalization. While they can generally produce satisfactory outcomes in repetitive scenarios, they cannot adapt to novel or dynamic environments (Table II) [47].
2) ***Machine Learning (ML) Techniques****.* Medium complexity and speed, tweakable and mostly reliable. These techniques exhibit greater flexibility in comparison to conventional algorithms. It is important to note that the algorithms trained are subject to the constraints imposed by supervised learning, which requires the use of training data [47].
3) ***Deep Learning (DL) Techniques****.* High complexity (i.e., prolonged training time to train, inconsistent performance). They are the most adaptable to changing image conditions and provide the most accurate results [47]. A strong limitation of the *ad hoc* UNet is the small size of the dataset. While DL models are mainly trained with hundreds or thousands of data [48], we provided this model with 101 images in the best case, and no previous context.

In this regard, the missing Validation Set is an added limitation. Even though it is mitigated through cross-validation, a bigger dataset size would be needed in future stages, so as to provide validation KPIs.

How masks are created automatically is quite relevant. The same Spheroid can be segmented in two completely different ways, depending on the algorithm (Fig 9). Otsu’s algorithm was chosen, as it selects the maximum variance to create the threshold.

The metrics are not to be compared with each other. Jaccard is much stricter than NCC. As a result, if the goal is to have good tracking and a deep understanding of how necrosis manifests in a spheroid, the mask-based models are recommended. On the other hand, if an image, or a gradient of less to more necrotic is sought, the other models comply.

Finally, performing standardized sensitivity analysis for image-to-image deep learning models remains complex and susceptible to interpretive bias. Instead of applying sensitivity analysis, we prioritize analyzing the underlying performance distributions, directly evaluating the advantages, disadvantages, and specific failure modes for each model architecture and image modality.4.3. Necrosis prediction using computer vision in CRC spheroids

The efficacy of the most basic and manual methods (Otsu/*Adaptive Thresholding*) stands out. In essence, most of the masks simply overlap the contour of the spheroid. For segmenting the whole spheroid (DAPI, not the necrotic zone) complex models do not outperform the basic fine-tuned algorithms. A specific or heavyweight solution like *WaterMeans* or *UNet* has proven to be more accurate for identifying necrotic areas, however.

In ML models, even though they cannot be simpler, the results are noteworthy. This indicates a clear correlation between necrosis and dark spots in the spheroids, provided this has been the only input in ML models. An example is illustrated in Fig 11, the Decision Tree Classifier, which processes a Brightfield image to a PI mask, thereby demonstrating its functionality.

UNet performance stands out from the rest of the techniques, yielding accurate and consistent results in almost all cases. A comparison of the image on the right to the binary solution (Fig 8, right) reveals a Jaccard similarity score of 0.88, indicative of a strong correlation between the two. Image-to-image methods also create images that closely resemble the stained spheroid. The regression U-Net model requires only the Brightfield image to generate an image that mimics a PI staining (Fig 12).

### 4.3. PC results

The results obtained with DAPI are similar to those observed in HT-29. In contrast, the situation with PI is quite different. Necrosis in Hs-766T is localized in small clusters (Fig 7), thereby leading to the failure of the algorithms designed to detect a central necrotic core. Consequently, the majority of the ML algorithms fail to identify the necrotic clusters. The algorithms optimize to black images that maximize the NCC. Thus, these models are unable to identify necrosis in Hs-766T spheroids. But DL algorithms behave better and produce patterns very similar to those shown by the masks. Still, it is very hard to get good performance because the dead cells appear in small clusters that are not easy to locate in the BF image. Notwithstanding, the images are encouraging, as evidenced by the DAPI and PI images in Fig 16.

### 4.4. Model performance comparison

All the models here depicted show comparable accuracy results (Fig 10). The methods that are oriented to solving this problem behave slightly better in terms of higher accuracy on average and smaller standard deviation.

**Fig 17:**
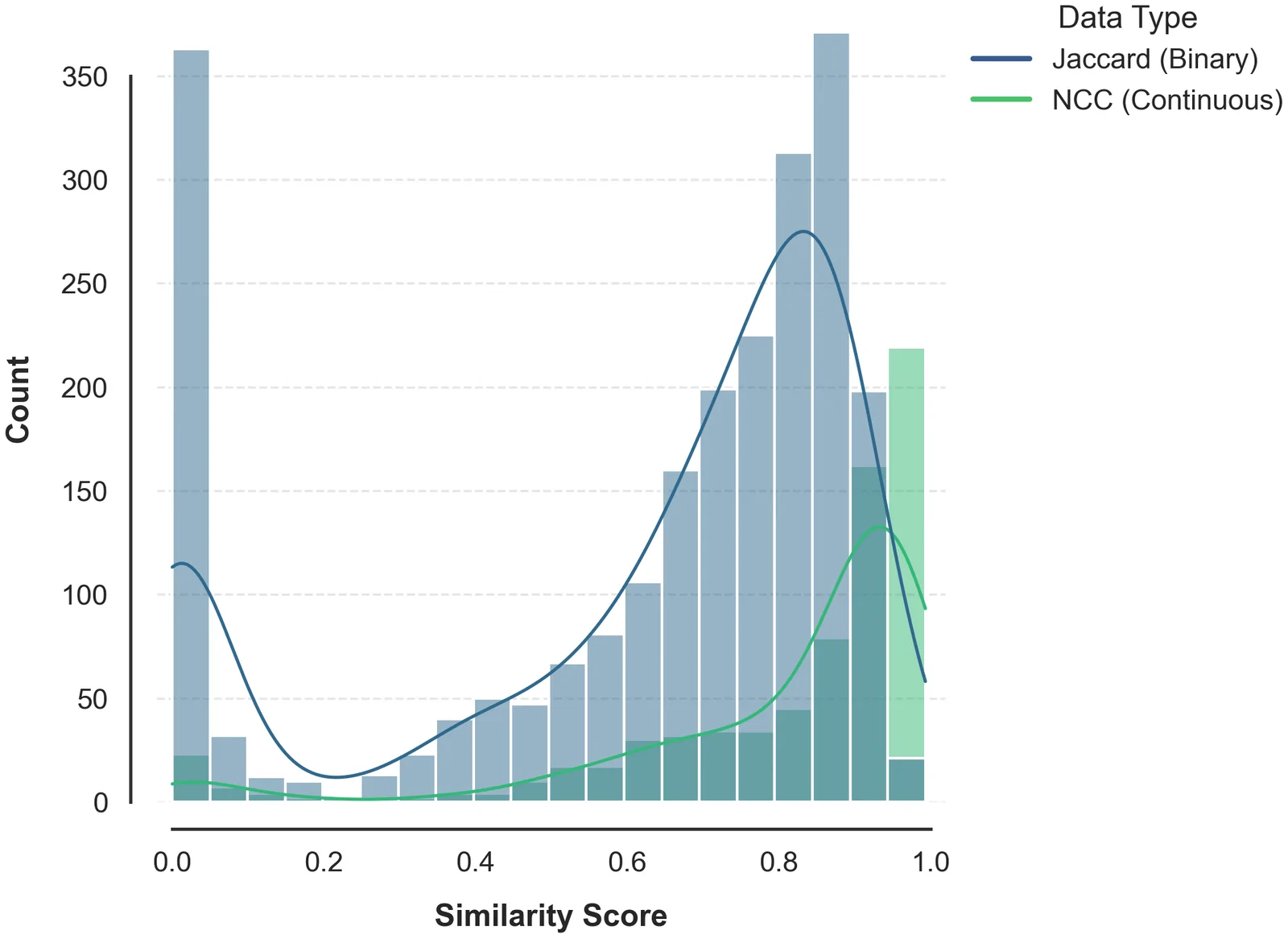
Assessment of the distribution of accumulated model success. It does not follow a normal distribution (Shapiro-Wilk p-value < 1e-5 for both datasets).

The score frequency must be evaluated as well, as it does not fully fit to a Gaussian distribution. Instead, the models predict with increasing accuracy. That is, the prediction models work very well for generic spheroid images, but do not excel in certain cases, namely Pancreatic with PI (Fig 17).

### 4.5. Generalization

As demonstrated in Table 2, generalized models significantly underperform compared to specialized models. This finding suggests that the different types of spheroids do not present the same pattern between dark areas in the spheroid and necrosis. Not only do cell-to-cell variations accumulate, but spheroid-to-spheroid factors, such as shape or structure, play a role. DL models are capable of distinguishing between PC and CRC spheroids, as their predictions of PC necrosis are less distinct. These models will therefore perform better in spheroids with a spatially coherent necrosis distribution, in opposition to PC’s scattered necrosis, where all models have struggled.

## 5 Conclusion

To our knowledge, this study represents a novel approach to spheroid necrosis modeling without

direct precedent in the field for comparative benchmarking. Our findings suggest that necrosis modeling in spheroids can be effectively achieved through brightfield microscopy 2D images. However, the model accuracy heavily depends on the specific morphology and cell line characteristics of the tumor spheroids. While PI and DAPI staining provided high-quality fluorescent validation of necrotic regions in both colorectal CRC and pancreatic cancer (PC) spheroids, performance varied by cancer type and the spheroid's morphology and structure. In the case of spheroids that exhibit concentrated core necrosis (CRC), segmentation efficiency reached 75% and regression techniques yielded up to 95% accuracy in the best scenarios.

The existence of a correlation between necrosis and the presence of dark spots in images has been substantiated by the application of machine learning (ML) models. Conversely, the most unfavorable outcome identified in the present study was observed in the pancreatic spheroids with PI. The nodular nature of Hs-766T spheroids has resulted in the manifestation of necrosis in small, widespread areas, which have proven challenging to detect by the machine learning models. The disparities observed over time and between cell lines are substantial, impeding the development of a universally applicable model for necrosis analysis in spheroid BF images. Nevertheless, in this work we successfully validate the use of DAPI and PI for characterizing necrotic regions. Building upon this, we introduced a fast, cost-efficient, and highly accurate machine learning framework to identify necrosis within HT-29 and Hs766-T cancer spheroids. Crucially, this system operates independently of fluorescent staining, relying exclusively on standard brightfield (BF) images. By providing a non-invasive digital inference, our approach offers a scalable, label – free alternative that opens new possibilities for cancer research and longitudinal drug screening.
