# Supplementary material for: Machine learning-supported identification of necrosis in colorectal and pancreatic cancer spheroids
Source: PLoS One. 2026 Sep 15;21(9):e0357972. doi: 10.1371/journal.pone.0357972 (PMC13577338; doi:10.1371/journal.pone.0357972)
Supplement: Supplementary file 1 [file pone.0357972.s002.pdf]

PLOS ONE Decision: Revision required [PONE-D-26-05059] - [EMID:44b1e5e7467fe892]

Please include the following items when submitting your revised manuscript:

- **A letter that responds to each point raised by the academic editor and reviewer(s).** You should upload this letter as a separate file labeled 'Response to Reviewers'.
- A marked-up copy of your manuscript that highlights changes made to the original version. You should upload this as a separate file labeled **'Revised Manuscript with Track Changes'.**
- An **unmarked version of your revised paper without tracked changes**. You should upload this as a separate file labeled 'Manuscript'.

- Please use the space provided to explain your answers to the questions above. You may also include additional comments for the author, including concerns about dual publication, research ethics, or publication ethics. (Please upload your review as an attachment if it exceeds 20,000 characters).

Please submit your revised manuscript by Jun 11 2026 11:59PM (Postponed to June 22 2026).

<u>Review 1 Comments to the Author:</u>

Reviewer #1:

This manuscript describes a machine learning– and deep learning–based framework to identify necrotic regions in colorectal (HT-29) and pancreatic (Hs766-T) cancer spheroids using only brightfield microscopy images. Fluorescence staining (DAPI, PI, TTC) is used to establish ground truth, and multiple computational approaches—ranging from classical image processing to U-Net architectures—are systematically evaluated.

The study is methodologically sound, transparently reported, and the conclusions are supported by the data presented. The work fits well within PLOS ONE's scope as a

methods- and tool-oriented contribution. Importantly, the authors clearly acknowledge limitations regarding generalizability, dataset size, and the inherent constraints of 2D imaging of 3D structures.

- We sincerely thank you for your time and the thoughtful feedback provided on our manuscript. Your comments are highly appreciated and have helped us refine our work. Please find our point-by-point responses to your comments below.

1) Clarify dataset composition: It would be helpful to include a concise summary (e.g., a short paragraph or table) specifying how many images/spheroids were used for training versus testing for each model type, especially for the U-Net experiments.

- The train/test split was clearly missing. We added a small clarification in the Methods - Machine Learning Heading:

1. "For these algorithms, the dataset was split into Train and Test groups, 70/30." Page 7, lines 191-192.
2. "A validation group was not created due to the small dataset size. To avoid overfitting, we did a 5-Fold Cross Validation." Page 8, lines 212-213
3. Completed in Table 1, where we show the spheroid breakdown.
4. Lastly, we appended some related content in the Discussion, at the end of 4.2.

"A strong limitation of the *ad hoc* UNet is the small size of the dataset. While DL models are mainly trained with hundreds or thousands of data, we provided this model with 101 images in the best case, and no previous context.
In this regard, the missing Validation Set is an added limitation. Even though it is mitigated through cross-validation, a bigger dataset size would be needed in future stages, so as to provide validation KPIs." Pages 14, lines 388-393

2) While the use of Jaccard similarity and normalized cross-correlation is appropriate, a brief justification for choosing these metrics (particularly NCC for regression-style outputs) could improve accessibility for non-specialist readers.

- As suggested by the reviewer 1, we extended and justified this section, next to the descriptions:

1. "Jaccard Similarity is ideal for binary data, as it is the ratio between the pixels the Prediction system got right and the total number of positive pixels. It is easy to implement and grants good intuition (Jaccard Similarity of 50% means the system got half of the necrotic pixels right)." Page 9, lines 248-250
2. "The second approach entails a comparison of whole images to obtain a percentage of similarity. The Normalized Coefficient of Correlation (NCC) was selected as the metric for evaluating the similarity between images. This coefficient, based on convolving one image over the other, ranges from -1 to 1 is fast, simple, and well-proven and ubiquitous in signal and image analysis. Additionally, it is quite intuitive to understand: two identical images have an NCC of 1, one image and its negative have a -1 NCC, and one image with random noise will have an NCC very close to 0. NCC has a flaw: it is not robust against rotations. In this study, we do not compare rotated spheroids; thus, this method is consistent." Page 9, lines 251-258
3. In addition, in the discussion: "The metrics are not to be compared with each other. Jaccard is much more strict than NCC. As a result, if the goal is to have good tracking and a deep understanding of how necrosis manifests in a spheroid, the mask-based models are recommended. On the other hand, if an

image, or a gradient of less to more necrotic is sought, the other models comply." Page 14, lines 397-400.

3) The manuscript is generally well written, but minor grammatical inconsistencies and occasional repetition in the Discussion could benefit from light editorial polishing.

- Thank you for this constructive suggestion. We have thoroughly reviewed and revised the entire manuscript to improve clarity, fix grammatical inconsistencies, and eliminate repetitive phrasing. We paid special attention to streamlining the Discussion section as requested. All minor grammatical and phrasing changes have been tracked in the attached version of the manuscript ("track changes" mode) for your convenience.

4) The discussion could be strengthened by slightly more explicit framing that the approach is best suited to spheroid models with spatially coherent necrosis, rather than implying future near-term generalization across tumor types.

- We find the reviewer 1 observation on-point, and so do the results indicate. We agree and have addressed the discussion - generalization header accordingly.

"These models will therefore perform better in spheroids with a spatially coherent necrosis distribution, in opposition to PC's scattered necrosis, where all models have struggled." Page 16, lines 445-446.

Review 2 Comments to the Author:

Reviewer #2:

This manuscript investigates whether necrosis in 3D cancer spheroids can be identified directly from brightfield microscopy images using computer vision and machine learning, thereby reducing the need for staining-based assays. The authors study two spheroid models, colorectal HT-29 and pancreatic Hs-766T, and use DAPI, PI, and TTC-based staining to generate reference signals for necrotic or viable regions. They then compare several traditional image-processing methods, machine-learning models, and U-Net-based deep learning approaches for predicting necrosis patterns from brightfield images alone. Please address the following questions for each section of the manuscript.

- We thank the reviewer for this accurate and comprehensive summary of our manuscript. We appreciate the time and effort invested in evaluating our work. Below, we provide detailed, point-by-point responses to each of the specific questions and comments raised.

Introduction

1. In the current Introduction, machine learning is barely introduced. It only appears implicitly at the very end when the authors state that they propose a “computer vision system” using brightfield images.

- We agree with the reviewer: there is a lack of references to CV in the introduction. We have added a small paragraph introducing it and underlining its role.

“Computer Vision (CV) has already proved useful in similar contexts. Given a spheroid image dataset, there are a plethora of supervised and unsupervised methods with a

wide span of complexity (traditional CV methods to Machine and Deep Learning).” Page 3, lines 64-66.

And at the end of the introduction: “..the present study explores possible Computer Vision systems for the automated detection of necrosis in cancer spheroids. The objective was to develop a cost-effective, fast, and precise automatic tool that segments necrosis in spheroids without the need for staining, relying exclusively on Brightfield (BF) images.” Page 3, lines 87-91.

2. The discussion of necrosis is brief and somewhat generic. It does not fully explain its broader role in the tumor microenvironment.

- We appreciate this valuable feedback. We agree that providing a deeper biological context enhances the effectiveness of our machine learning and deep learning based framework to identify necrotic regions in colorectal (HT-29) and pancreatic (Hs766-T) cancer spheroids using only brightfield microscopy images.

Accordingly, we have expanded the 'Discussion' section significantly to detail the broader role of necrosis within the tumour microenvironment. Specifically, we have added text that addresses how necrosis drives chronic inflammation, promotes angiogenesis and contributes to tumour progression and therapy resistance. Page 3, lines 71-79.

3. The manuscript lacks a clear discussion of previously established methods for detecting necrotic regions.

- We appreciate the reviewer's feedback. We have revised the manuscript to include a dedicated discussion of the pros and cons of existing state-of-the-art and traditional methods for necrosis detection relative to our proposed brightfield-based machine

learning approach. These changes can be viewed in the attached manuscript, the text above was added to the introduction section of the manuscript:

“An important process related to metastasis is necrosis, there is a large body of evidence that links the presence of necrosis inside solid tumors as colorectal cancer and pancreatic cancer as a strong indicator of a highly aggressive cancer and poor patient prognosis. Dead cells cannot metastasize but alter the tumoral environment for the alive neighboring cancer cells to escape and spread throughout the body. All previously mentioned, this underscores the critical importance of cell death control and monitoring in cancer therapy. Actual methods of detecting necrosis in multicellular tumoral spheroids, such as standard fluorescence live/dead staining followed by confocal microscopy, histological sections followed by haematoxylin and eosin microscopic analysis, optical coherence tomography (OCT), and Light sheet fluorescence microscopy to map exposed F-actin in necrotic cores, are often expensive, time consuming and destructive to the sample. To overcome these limitations….” Page 3, Lines 77 to 87

Methodology

1. How data were split into training, validation, and test sets. Whether images from the same spheroid appear in multiple sets (risk of data leakage). Whether evaluation was done using cross-validation or a single split. Without this, the reported performance (e.g., ~90% correlation) cannot be considered reliable.

- Thank you for raising these critical points regarding our validation strategy. We completely agree that ensuring data integrity and preventing data leakage is essential for the reliability of the reported performance. We have addressed these points as follows:

Data Leakage Prevention: There is no risk of data leakage across the sets. Spheroids were imaged and processed immediately following staining, and because the propidium iodide (PI) stain is toxic (which would artificially expand the necrotic area over time), each spheroid was imaged exactly once. No technical replicates or sequential images of the same spheroid were used. Thus, images from the same spheroid never appear in multiple sets.

Cross-Validation Strategy: To maximize the utility of our dataset while ensuring robust evaluation, we only used K-Fold Cross-validation in DL, and was not mentioned, as you point out. We apologize for omitting this detail in the original manuscript draft. We have updated the Materials and Methods section to explicitly state this workflow:

“A validation group was not created due to the small dataset size. To avoid overfitting, we did a 5-Fold Cross Validation.” Page 8, lines 212-213

2. Although ~101 spheroids are mentioned, the methodology does not clarify: How many images per condition were used for training vs testing. Whether datasets are balanced between: CRC vs pancreatic early vs late necrosis stages.

- The train/test split was clearly missing. We added a small clarification in the Methods - Machine Learning Heading, completed it in Table 1, where we show the spheroid breakdown, and appended some related content in the Discussion, at the end of 4.2.

The small dataset has been a constant constraint in this study. We opted for a dataset with all spheroids of CRC to train the models. This is because we assumed that the necrosis would evolve similarly across time, and not be a parameter to input to the models.

3. The study relies on DAPI/PI staining as “ground truth,” but: The exact criteria for defining necrotic pixels is not clearly described

It is unclear how: thresholds were chosen and binary masks were validated. No discussion of biological limitations (necrosis vs apoptosis overlap).This is a core issue because ML models depend entirely on label quality.

- We thank the reviewer for highlighting this point. We have added a detailed explanation in the **Methods - Solution Metric section (pag.8 line 228)** to clarify our approach. Specifically, we utilized **Otsu's method** to eliminate the need for manual threshold selection, which could introduce bias or alter the results. Otsu's method automatically separates the mask and the background by maximizing inter-class variance, ensuring an objective and reproducible segmentation process.

To ensure a uniform and unbiased binarization process, binary masks are automatically generated from the stained images using Otsu's thresholding method. While a fixed manual threshold could be applied, Otsu's algorithm dynamically calculates the optimal threshold by maximizing the inter-class variance between the foreground (1) and background (0) pixels. This approach minimizes subjectivity and yields higher-quality masks that more accurately represent the true staining patterns.

4. For ML and DL models, the following are missing: Hyperparameters (learning rate, epochs, batch size, etc.). Training procedure (optimizer, loss function). Architecture details for U-Net (depth, filters, layers). Regularization methods (dropout, augmentation). Hardware/software environment

- We have included the details of both models in a list in Materials and Methods.

"Logistic Regression - Scikit Learn: C=1.0, l1_ratio=0.0, dual=False, tol=0.0001, fit_intercept=True, intercept_scaling=1, class_weight=None, random_state=None, solver='lbfgs', max_iter=1000

Decision Tree Classifier - Scikit Learn: criterion='gini', splitter='best', max_depth=None, min_samples_split=2, min_samples_leaf=1, min_weight_fraction_leaf=0.0, max_features=None, random_state=None, max_leaf_nodes=None, min_impurity_decrease=0.0, class_weight=None, ccp_alpha=0.0, monotonic_cst=None

Decision Tree Regressor - Scikit Learn: criterion='squared_error', splitter='best', max_depth=None, min_samples_split=2, min_samples_leaf=1, min_weight_fraction_leaf=0.0, max_features=None, random_state=None, max_leaf_nodes=None, min_impurity_decrease=0.0, ccp_alpha=0.0, monotonic_cst=None" Page 7, lines 193-205

For DL, we also included a simple figure showing the architecture, plus hyperparameter and layer description:

"The UNet we implemented has a 5-layer architecture. Each layer comprises:

ReLU-activated, same padding Convolution.

Dropout of 0.1 in upper levels, 0.2 in medium, 0.3 in lower levels, to favor smaller features.

ReLU-activated, same padding Convolution.

Average (binary) or Max (regression) Pooling Layer.

And was trained using these hyperparameters:

Optimizer: Adam, Learning Rate 1e-5

Epochs: 70

Batch size: 1

Loss function: Binary Cross Entropy

Metric: Binary Accuracy (binary), Mean Squared Error (regression)" Page 8, line 213-224.

Referring to software and hardware environments, all models (including the UNets) are rather lightweight, and can be trained even without a GPU (would not recommend). Therefore, we've prepared them to run in the free version of Google Colab Notebook, so that it is easy to share and to train for anyone. Per se, there is no "minimum hardware requirements", but the less powerful the hardware, the more time it will take to train the models (specially the UNet - recommend 16 Gb RAM, GPU).

5. While metrics are mentioned (Jaccard, NCC), the following are missing: How metrics are computed (per image? averaged?)

- Thank you for pointing out this omission. We agree that the exact method of metric computation should be explicitly stated.

Metrics are computed per image, i.e.: we run the metric (Jaccard or NCC) of the predicted image/mask vs the real staining image/mask.

Whether results are reported with:

standard deviation

confidence intervals

Statistical comparison between models

- We have appended Figures 10 and 17, and expanded table 2 to show some statistical analysis. Namely, we've added the standard deviations and assessed independence between Models and Dyes through unpaired T-Test and ANOVA. P-Values have been indicated in the Figure description.

“Figure 10. Model performance breakdown. Left: Mask-based models. One-way ANOVA was carried out to assess inter-model ($p<0.01$) and inter-dye ($p<0.01$) independence. Right: Regression-based models. T-Test was carried out to assess inter-model ($p<0.01$) and inter-dye ($p<0.01$) independence.” Page 11, lines 309-312.

6. The manuscript includes multiple algorithms but lacks:

A clearly defined baseline method

Justification for choosing specific algorithms

Comparison to existing published approaches

-Thank you for this valuable suggestion. We have expanded our literature review to include new references to studies involving spheroids and computer vision (see Page 3, line 64). However, they do not cover exactly the same morphological segmentation, therefore not all models utilized in our study are featured in those papers. Likewise, to the best of our knowledge no established, direct benchmark currently exists to compare against for this specific task. We have expanded the motivation for each algorithm in Materials and methods:

“Otsu’s algorithm: this algorithm converts a grayscale image into a two-color image (i.e., binary mask) using the pixel-value threshold that ensures maximum variance. It is very fast and shows efficacy in many contexts. We used Otsu’s algorithm to 1) Automatically Generate Masks from DAPI and PI Images, 2) Delineate the Brightfield spheroid image to match DAPIs output.

Watershed algorithm. It is a classic segmentation algorithm, which can be considered a good benchmark for most computer vision applications of this kind. It is important to note that this algorithm necessitates a certain degree of pre-processing to identify specific features of the object.

Logistic Regression. Machine-learning technique that has been developed for analyzing binary data. This algorithm is supervised, thus necessitating a training dataset. The trained model predicts the probability of a new value being.

Decision Tree Classifier and Regressor. It is a machine learning algorithm that employs a tree-based structure for data visualization and the decision-making process. This is trained to generate a series of straightforward decisions that categorize or predict a probability.

U-Net. The only Deep Learning algorithm tested. It consists of two Convolutional Neural Networks in a symmetric architecture, such that the first one works as an encoder and the second one as a decoder. The implementation of the network was conducted without same-dimensional space feedback.

Watershed after K-Means (K=3) (referred to as WaterMeans). K-Means is an unsupervised machine-learning clustering algorithm that divides any group of data into the indicated number of clusters . In this work, K was set to 3 to divide the spheroid into Necrotic, Quiescent, and Proliferative zones. We generate an image in which every pixel is a binary mask (Figure 4). The Watershed algorithm attempts to expand from this point in all directions until it finds a border." Page 6, lines 154-176

7. The methodology does not clearly describe: Biological replicates vs technical replicates, Variability between experiments/days

- In our study, we utilize Biological replicates consisting of independently cultured spheroids. This approach allows us to accurately capture biological variance and generalize our findings to a broader population, which is the statistically rigorous method for testing our hypothesis.

Controls for staining consistency.

- To ensure staining consistency and eliminate inter-assay variability, the entire cohort of independent spheroids was processed, stained, and imaged simultaneously in a single batch. We maintained a strictly constant concentration of the staining agent across all samples, and all imaging was performed using fixed exposure settings (Exposure Value, EV) to prevent any instrument-introduced variance. Because the spheroids were analyzed under identical technical conditions, the variation observed in our data purely reflects true biological diversity rather than technical or procedural artifacts.

Result

1. Although some metrics are mentioned (e.g., Jaccard similarity, normalized correlation), the Results section does not provide:
Mean ± standard deviation

Confidence intervals

Statistical significance (e.g., p-values)

- We thank the reviewer for the comments, incorporating descriptive statistics strengthens the rigor of our results.

To address this, we have expanded Table2 to include standard deviations and appended Figures 10 and 17 to present formal statistical analyses. Specifically, we employed one-way ANOVA and unpaired T-Test and to evaluate significant variations across different Models and Dyes. The P-Values are indicated in Figure 10 description.

"Figure 10. Model performance breakdown. Left: Mask-based models. One-way ANOVA was carried out to assess significant inter-model ($p<0.01$) and inter-dye ($p<0.01$) differences . Right: Regression-based models. T-Test was carried out to assess significant inter-model ($p<0.01$) and inter-dye ($p<0.01$) differences ." Page 11, line 309-312.

2. Multiple approaches are tested (traditional CV, ML, DL), but:

There is no structured comparison

No clear statement of: Which method performs best under what conditions

Table II is referenced, but not well explained in the text

- We thank the reviewer and agree. We have extended Table 2 and appended Figure 10 to do a comparison among all models and conditions tested.

3. The Results section does not clearly state:

How many images were used per experiment

Whether results are from:

training data

validation data

independent test data

- Unsupervised models to not need Train/Test split -> The full dataset was used to validate

- ML Supervised models were split in train and test -> Results belong to test split

- KFolds for DL (supervised) models

4. There is no:

Cross-validation

Repeated experiments

Sensitivity analysis

- We appreciate the reviewer's focus on the robustness of our evaluation. We have addressed these concerns as follows:

1. Cross-Validation: As detailed in our response to Question 1 and updated in the Methodology section, a rigorous cross-validation framework was utilized for the deep learning models to ensure generalizability and prevent overfitting.
2. Repeated Experiments: We have clarified the experimental protocol in the revised manuscript. Biological replicates were performed across samples. Samples exhibiting poor dye infiltration under microscopic quality control (e.g., TTC staining inefficacy) were excluded pre-analysis based on predefined quality control criteria to ensure data integrity, rather than post-hoc data selection.
3. Sensitivity Analysis: Because our primary objectives and conclusions rely on global, image-wise metrics, we omitted the highly granular, pixel-wise sensitivity analysis to maintain manuscript conciseness and clarity. Instead, we focused on studying the distribution of the results and the reasons behind it, elaborating on upsides and downsides of each model and image type.

5. HT-29 spheroids are analyzed across multiple days (including later stages 18 days) Hs-766T spheroids appear to have fewer or shorter time points (e.g., around 7–14 days). But the time coverage is not consistent between the two cell lines.

- We thank the reviewer for noting this difference in time coverage between the two cell lines.

The variance in timelines is primarily due to the distinct biological characteristics, proliferation rates and spheroid structural integrity of the study cell lines. The HT-29 (colorectal cancer spheroids) maintain structural integrity and viability up to day 18, however, Hs-766T (pancreatic cancer spheroids) progress more rapidly towards structural degradation, limiting reliable analysis beyond days 7 to 14.

Rather than standardising both lines to the lowest common denominator (which would result in the discarding of valuable late-stage data for the HT-29 line), we deliberately opted to include the full viable lifespan of both spheroid types.

We have added a note to Section **Materials and Methods** of the revised manuscript to explicitly clarify these biological differences and justify the asymmetrical timelines (Page 5, lines 137-143).

Discussion:

1. The discussion does not adequately compare results with previous studies

- Previous studies have not been included in the discussion, to avoid noise, as they do not tackle the same problem, but similar ones (e.g. Regression to estimate spheroid viability: Image to Number, not Image to Mask/Image as in this project).

- Deep Learning for Deep Learning for Predicting Spheroid Viability: Novel Convolutional Neural Network Model for Automating Quality Control for Three-Dimensional Bioprinting Non-invasive quantification of viability in liver spheroids using deep learning - PMC
- Inverse Protocol Prediction from Spheroid Microscopy Imaging via Morphology-Aware Structured LearningPredicting Spheroid Viability: Novel Convolutional

Neural Network Model for Automating Quality Control for Three-Dimensional Bioprinting

- Non-invasive quantification of viability in liver spheroids using deep learning - PMC
- Inverse Protocol Prediction from Spheroid Microscopy Imaging via Morphology-Aware Structured Learning

2. Although some limitations are hinted, the section lacks a structured and explicit **limitations paragraph.**

- Thank you for this constructive recommendation. We agree that a structured and explicit discussion of the study's constraints strengthens the transparency and rigor of our work. In accordance with your feedback we have added a dedicated section (4.2 Limitations) within the discussion section. For your information, the newly added text is reproduced below:

“4.2. Limitations.

Besides the above-mentioned pancreatic spheroid challenge, this study is marked by two main constraints: the size of the dataset and the extrapolation of three-dimensional features from two-dimensional images. Other limitations include the number of dyes (a second necrosis stain like indocyanine green can provide a second source of data), and the inability to differentiate whether the dead cells are apoptotic.

In this study, we examine three primary types of algorithms, each of which exhibits notable efficacy in specific contexts.

1) Traditional Computer Vision Algorithms. They are characterized by their cost-effectiveness, speed, adaptability, and reliability. They are unsupervised, and not subject to the constraints imposed by supervised learning. However, they exhibit

limited flexibility and generalization. While they can generally produce satisfactory outcomes in general, repetitive scenarios, they face challenges in adapting to novel or dynamic environments, but they struggle to adapt (Table II).

2) Machine Learning (ML) Techniques. Medium complexity and speed, tweakable and mostly reliable. These techniques exhibit greater flexibility in comparison to conventional algorithms. It is important to note that the algorithms trained are subject to the constraints imposed by supervised learning, which requires the use of training data.

3) Deep Learning (DL) Techniques. High complexity (i.e., prolonged training time to train, inconsistent performance). They are the most adaptable to changing image conditions and provide the most accurate results. A strong limitation of the ad hoc UNet is the small size of the dataset. While DL models are mainly trained with hundreds or thousands of data, we provided this model with 101 images in the best case, and no previous context.

In this regard, the missing Validation Set is an added limitation. Even though it is mitigated through cross-validation, a bigger dataset size would be needed in future stages, so as to provide validation KPIs.

How masks are created automatically is quite relevant. The same Spheroid can be segmented in two completely different ways, depending on the algorithm (Figure 9 and Figure 8). Otsu's algorithm was chosen, as it selects the maximum variance to create the threshold.

The metrics are not to be compared with each other. Jaccard is much more strict than NCC. As a result, if the goal is to have good tracking and a deep understanding of how necrosis manifests in a spheroid, the mask-based models are recommended. On the

other hand, if an image, or a gradient of less to more necrotic is sought, the other models comply.” Pages 13-14, lines 369-400

3. The discussion mentions differences between HT-29 and Hs-766T spheroids, but: not deeply explore why necrosis patterns differ biologically.

- We thank the reviewer for this excellent suggestion. We agree that deeply exploring the biological divergence between these two cell lines adds substantial value to the paper and explains why the computer vision models perform differently on them.

We have expanded the Discussion section to detail the biological mechanisms driving these distinct necrosis patterns. Specifically, we have added a discussion on how the tightly packed, cohesive architecture of colorectal HT-29 spheroids creates a steep, predictable oxygen/nutrient gradient leading to a *spatially coherent* necrotic core. Unlike the uniform, compact cellular packing typical of colorectal HT-29 models, pancreatic adenocarcinoma cells retain intrinsic glandular features. These include the tendency to form irregular duct-like structures and micro-lumens, as well as multi-centric clusters. This inherent architectural heterogeneity disrupts the formation of a singular, linear nutrient gradient, instead leading to localised micro-environments and the scattered necrosis observed in our results. A paragraph about this topic can be found in the manuscript (Page 13, lines 365-368)

4. The manuscript suggests usefulness for drug screening, but does not clearly explain

- We agree that clarifying the practical utility of our method in drug screening workflows will significantly strengthen the manuscript's translational value. Traditional staining methods are destructive, expensive, and time-consuming, whereas our method allows for non-destructive, real-time, kinetic monitoring of drug efficacy on the exact same

spheroids over time. The discussion in the following paragraph was incorporated into the text:

"Nevertheless, in this work we successfully validate the use of DAPI and PI for characterizing necrotic regions. Building upon this, we introduced a fast, cost-efficient, and highly accurate machine learning framework to identify necrosis within HT-29 and Hs766-T cancer spheroids. Crucially, this system operates independently of fluorescent staining, relying exclusively on standard brightfield (BF) images. By providing a non-invasive digital inference, our approach offers a scalable, label - free alternative that opens new possibilities for cancer research and longitudinal drug screening." Page 16-17, lines 463-469.
