# Supplementary material for: Machine learning-supported identification of necrosis in colorectal and pancreatic cancer spheroids
Source: PLoS One. 2026 Sep 15;21(9):e0357972. doi: 10.1371/journal.pone.0357972 (PMC13577338; doi:10.1371/journal.pone.0357972)
Supplement: Supplementary file 2 [file pone.0357972.s003.docx]

**Reviewer’s answers**

We thank the reviewers for their thoughtful feedback and constructive suggestions. We have carefully addressed each of the remaining remarks to strengthen our manuscript. Bellow, we provide point by point responses outlining the revisions made on the text:

1.**The comparison with previous studies could be further strengthened by providing a more critical discussion of related machine learning and image-analysis approaches.**

We appreciate this suggestion since this was only partially addressed in the previous manuscript version. To provide a more thorough and critical context, we have restructured and extended the paragraph in the Introduction surveying state of the art (SOTA) computer vision methods applied to spheroids (Page 3, lines 64-70):

*“Computer Vision (CV) has proven effective in similar contexts; however, existing state of the art methods focuses primarily on studying spheroid viability (extracted as a ratio from 0 to 1)^13,14^, or extracting growth KPIs^15^ from single Brightfield images using mostly Deep Learning Methods.*

*While these approaches offer valuable methodological insights, they address fundamentally different task formulations. Specifically, image to scalar regression. Our framework, nonetheless, requires image to image (or image to mask) translation models. Consequently, direct benchmarking against these pre-existing models was not methodologically feasible.”*

**2. In addition, clearer reporting of the exact numbers of images used for the training and testing datasets would improve transparency and reproducibility.**

We agree that precise reporting of dataset partitioning is essential for reproducibility. We have updated the Material and Methods section to clearly indicate when a split is needed and the split proportions. The exact number of replicates allocated to each split is provided in Table I (Page 7, lines 181-184):

*“For classical Machine Learning algorithms (Logistic Regression and Decision Trees), a 70/30 train/test split was applied. The exact number of spheroid replicates corresponding to these splits is detailed in Table I. For Deep Learning architectures, a 5-fold cross-validation scheme was implemented to mitigate overfitting and ensure robust performance evaluation.”*

**3. Although the statistical analysis has been strengthened, reporting confidence intervals would provide additional support for the robustness of the findings.**

We strongly agree with the reviewer, especially as it was reported in the discussion that the score distribution is not always gaussian, where traditional standard deviation bounds (e.g., 0.911 ± 0.118) can be misleading. Therefore, we have replaced all the standard deviation metrics with non-parametric Bootstrap Mean-based 95% confidence intervals. We believe this provides a significantly more accurate representation of our results. The updated metrics have been incorporated through Table II (pages 11-12, line 316).

**4. Finally, while the omission of a sensitivity analysis is reasonably justified, this limitation should remain clearly acknowledged in the Discussion.**

We appreciate this point. To ensure complete transparency, we have added an explicit statement acknowledging this limitation at (page 16, lines 411-414):

*“Finally, performing standardized sensitivity analysis for image-to-image deep learning models remains complex and susceptible to interpretive bias. Instead of applying sensitivity analysis, we prioritize analysing the underlying performance distributions, directly evaluating the advantages, disadvantages, and specific failure modes for each model architecture and image modality.”*
