# Supplementary material for: Machine learning-supported identification of necrosis in colorectal and pancreatic cancer spheroids
Source: PLoS One. 2026 Sep 15;21(9):e0357972. doi: 10.1371/journal.pone.0357972 (PMC13577338; doi:10.1371/journal.pone.0357972)
Supplement: Peer review history

# Peer review: Decision Letter 0

**DOI:** 10.1371/journal.pone.0357972.r001

**Authors:** Xiaozhe Han

27 Apr 2026

Dear Dr. Prado López,

Thank you for submitting your manuscript to PLOS ONE. After careful consideration, we feel that it has merit but does not fully meet PLOS ONE’s publication criteria as it currently stands. Therefore, we invite you to submit a revised version of the manuscript that addresses the points raised during the review process.

Please submit your revised manuscript by Jun 11 2026 11:59PM. If you will need more time than this to complete your revisions, please reply to this message or contact the journal office at plosone@plos.org. When you're ready to submit your revision, log on to https://www.editorialmanager.com/pone/ and select the 'Submissions Needing Revision' folder to locate your manuscript file.

- A letter that responds to each point raised by the academic editor and reviewer(s). You should upload this letter as a separate file labeled 'Response to Reviewers'.
- A marked-up copy of your manuscript that highlights changes made to the original version. You should upload this as a separate file labeled 'Revised Manuscript with Track Changes'.
- An unmarked version of your revised paper without tracked changes. You should upload this as a separate file labeled 'Manuscript'.

If you would like to make changes to your financial disclosure, please include your updated statement in your cover letter. Guidelines for resubmitting your figure files are available below the reviewer comments at the end of this letter.

If applicable, we recommend that you deposit your laboratory protocols in protocols.io to enhance the reproducibility of your results. Protocols.io assigns your protocol its own identifier (DOI) so that it can be cited independently in the future. For instructions see: https://journals.plos.org/plosone/s/submission-guidelines#loc-laboratory-protocols. Additionally, PLOS ONE offers an option for publishing peer-reviewed Lab Protocol articles, which describe protocols hosted on protocols.io. Read more information on sharing protocols at https://plos.org/protocols?utm_medium=editorial-email&utm_source=authorletters&utm_campaign=protocols.

As the corresponding author, your ORCID iD is verified in the submission system and will appear in the published article. PLOS supports the use of ORCID, and we encourage all coauthors to register for an ORCID iD and use it as well. Please encourage your coauthors to verify their ORCID iD within the submission system before final acceptance, as unverified ORCID iDs will not appear in the published article. *Only*  the individual author can complete the verification step; PLOS staff *cannot*  verify ORCID iDs on behalf of authors.

We look forward to receiving your revised manuscript.

Kind regards,

Xiaozhe Han, D.M.D., Ph.D.

Academic Editor

PLOS One

**Journal Requirements:**

1. When submitting your revision, we need you to address these additional requirements.

Please ensure that your manuscript meets PLOS ONE's style requirements, including those for file naming. The PLOS ONE style templates can be found at

https://journals.plos.org/plosone/s/file?id=wjVg/PLOSOne_formatting_sample_main_body.pdf and

https://journals.plos.org/plosone/s/file?id=ba62/PLOSOne_formatting_sample_title_authors_affiliations.pdf

2. Thank you for stating in your Funding Statement:

This work was partially supported by funding through the IRA-SME project ELEVATE (FFG Grant No. 895365) to S.P-L.

Please provide an amended statement that declares *all* the funding or sources of support (whether external or internal to your organization) received during this study, as detailed online in our guide for authors at http://journals.plos.org/plosone/s/submit-now.  Please also include the statement “There was no additional external funding received for this study.” in your updated Funding Statement.

Please include your amended Funding Statement within your cover letter. We will change the online submission form on your behalf.

3. Thank you for stating the following financial disclosure:

This work was partially supported by funding through the IRA-SME project ELEVATE (FFG Grant No. 895365) to S.P-L.

Please state what role the funders took in the study.  If the funders had no role, please state: "The funders had no role in study design, data collection and analysis, decision to publish, or preparation of the manuscript."

If this statement is not correct you must amend it as needed.

Please include this amended Role of Funder statement in your cover letter; we will change the online submission form on your behalf.

4. Thank you for stating the following in the Acknowledgments Section of your manuscript:

The authors acknowledge TU Wien Bibliothek for financial support through its Open Access Funding Program. This work was partially supported by funding through the IRA-SME project ELEVATE (FFG Grant No. 895365) to S.P-L.

The code is publicly available: https://github.com/hibiscus22/NecrosisDetector/.

We note that you have provided funding information that is not currently declared in your Funding Statement. However, funding information should not appear in the Acknowledgments section or other areas of your manuscript. We will only publish funding information present in the Funding Statement section of the online submission form.

Please remove any funding-related text from the manuscript and let us know how you would like to update your Funding Statement. Currently, your Funding Statement reads as follows:

This work was partially supported by funding through the IRA-SME project ELEVATE (FFG Grant No. 895365) to S.P-L.

Please include your amended statements within your cover letter; we will change the online submission form on your behalf.

5. Please note that your Data Availability Statement is currently missing the repository name. If your manuscript is accepted for publication, you will be asked to provide these details on a very short timeline. We therefore suggest that you provide this information now, though we will not hold up the peer review process if you are unable.

6. Please include your full ethics statement in the ‘Methods’ section of your manuscript file. In your statement, please include the full name of the IRB or ethics committee who approved or waived your study, as well as whether or not you obtained informed written or verbal consent. If consent was waived for your study, please include this information in your statement as well.

7. Please include your tables as part of your main manuscript and remove the individual files. Please note that supplementary tables (should remain/ be uploaded) as separate "supporting information" files"

8. If the reviewer comments include a recommendation to cite specific previously published works, please review and evaluate these publications to determine whether they are relevant and should be cited. There is no requirement to cite these works unless the editor has indicated otherwise. 

Reviewers' comments:

**Comments to the Author**

1. Is the manuscript technically sound, and do the data support the conclusions?

Reviewer #1: Yes

Reviewer #2: No

**********

2. Has the statistical analysis been performed appropriately and rigorously?

Reviewer #1: Yes

Reviewer #2: No

**********

3. Have the authors made all data underlying the findings in their manuscript fully available?

The PLOS Data policy

Reviewer #1: Yes

Reviewer #2: No

**********

4. Is the manuscript presented in an intelligible fashion and written in standard English?

Reviewer #1: Yes

Reviewer #2: No

**********

**Reviewer #1:** This manuscript describes a machine learning– and deep learning–based framework to identify necrotic regions in colorectal (HT‑29) and pancreatic (Hs766‑T) cancer spheroids using only brightfield microscopy images. Fluorescence staining (DAPI, PI, TTC) is used to establish ground truth, and multiple computational approaches—ranging from classical image processing to U‑Net architectures—are systematically evaluated.

The study is methodologically sound, transparently reported, and the conclusions are supported by the data presented. The work fits well within PLOS ONE’s scope as a methods- and tool-oriented contribution. Importantly, the authors clearly acknowledge limitations regarding generalizability, dataset size, and the inherent constraints of 2D imaging of 3D structures.

1) Clarify dataset composition: It would be helpful to include a concise summary (e.g., a short paragraph or table) specifying how many images/spheroids were used for training versus testing for each model type, especially for the U‑Net experiments.

2) While the use of Jaccard similarity and normalized cross-correlation is appropriate, a brief justification for choosing these metrics (particularly NCC for regression-style outputs) could improve accessibility for non-specialist readers.

3) The manuscript is generally well written, but minor grammatical inconsistencies and occasional repetition in the Discussion could benefit from light editorial polishing.

4) The Discussion could be strengthened by slightly more explicit framing that the approach is best suited to spheroid models with spatially coherent necrosis, rather than implying future near-term generalization across tumor types.

**Reviewer #2:**  This manuscript investigates whether necrosis in 3D cancer spheroids can be identified directly from brightfield microscopy images using computer vision and machine learning, thereby reducing the need for staining-based assays. The authors study two spheroid models, colorectal HT-29 and pancreatic Hs-766T, and use DAPI, PI, and TTC-based staining to generate reference signals for necrotic or viable regions. They then compare several traditional image-processing methods, supervised machine-learning models, and U-Net-based deep learning approaches for predicting necrosis patterns from brightfield images alone. Please address the following questions for each section of the manuscript

Introduction

1. In the current Introduction, machine learning is barely introduced. It only appears implicitly at the very end when the authors state that they propose a “computer vision system” using brightfield images.

2. The discussion of necrosis is brief and somewhat generic. It does not fully explain its broader role in the tumor microenvironment

3. The manuscript lacks a clear discussion of previously established methods for detecting necrotic regions.

Methodology

1. How data were split into training, validation, and test sets. Whether images from the same spheroid appear in multiple sets (risk of data leakage). Whether evaluation was done using cross-validation or a single split. Without this, the reported performance (e.g., ~90% correlation) cannot be considered reliable.

2. Although ~101 spheroids are mentioned, the methodology does not clarify: How many images per condition were used for training vs testing. Whether datasets are balanced between: CRC vs pancreatic early vs late necrosis stages

3. The study relies on DAPI/PI staining as “ground truth,” but:

The exact criteria for defining necrotic pixels is not clearly described

It is unclear how: thresholds were chosen and binary masks were validated. No discussion of biological limitations (necrosis vs apoptosis overlap)

This is a core issue because ML models depend entirely on label quality.

4. For ML and DL models, the following are missing:

Hyperparameters (learning rate, epochs, batch size, etc.)

Training procedure (optimizer, loss function)

Architecture details for U-Net (depth, filters, layers)

Regularization methods (dropout, augmentation)

Hardware/software environment

5. While metrics are mentioned (Jaccard, NCC) , the following are missing:

How metrics are computed (per image? averaged?)

Whether results are reported with:

standard deviation

confidence intervals

Statistical comparison between models

6. The manuscript includes multiple algorithms but lacks:

A clearly defined baseline method

Justification for choosing specific algorithms

Comparison to existing published approaches

7. The methodology does not clearly describe:

Biological replicates vs technical replicates

Variability between experiments/days

Controls for staining consistency

Result

1. Although some metrics are mentioned (e.g., Jaccard similarity, normalized correlation), the Results section does not provide:

Mean ± standard deviation

Confidence intervals

Statistical significance (e.g., p-values)

2. Multiple approaches are tested (traditional CV, ML, DL), but:

There is no structured comparison

No clear statement of:

which method performs best

under what conditions

Table II is referenced, but not well explained in the text

3. The Results section does not clearly state:

How many images were used per experiment

Whether results are from:

training data

validation data

independent test data

4. There is no:

Cross-validation

Repeated experiments

Sensitivity analysis

5. HT-29 spheroids are analyzed across multiple days (including later stages 18 days)

Hs-766T spheroids appear to have fewer or shorter time points (e.g., around 7–14 days). But the time coverage is not consistent between the two cell lines.

Disscussion:

1. The discussion does not adequately compare results with previous studies

2. Although some limitations are hinted, the section lacks a structured and explicit limitations paragraph.

3. The discussion mentions differences between HT-29 and Hs-766T spheroids, but: not deeply explore why necrosis patterns differ biologically

4. The manuscript suggests usefulness for drug screening, but does not clearly explain

**********

what does this mean?). If published, this will include your full peer review and any attached files.

If you choose “no”, your identity will remain anonymous but your review may still be made public.

**Do you want your identity to be public for this peer review?**  For information about this choice, including consent withdrawal, please see our Privacy Policy

Reviewer #1: No

Reviewer #2: **Yes:** Elaheh Dalir Abdolahinia

**********

[NOTE: If reviewer comments were submitted as an attachment file, they will be attached to this email and accessible via the submission site. Please log into your account, locate the manuscript record, and check for the action link "View Attachments". If this link does not appear, there are no attachment files.]

To ensure your figures meet our technical requirements, please review our figure guidelines: https://journals.plos.org/plosone/s/figures

You may also use PLOS’s free figure tool, NAAS, to help you prepare publication quality figures: https://journals.plos.org/plosone/s/figures#loc-tools-for-figure-preparation.

NAAS will assess whether your figures meet our technical requirements by comparing each figure against our figure specifications.

# Author comment: Author response to Decision Letter 1

**DOI:** 10.1371/journal.pone.0357972.r002

26 Jun 2026

Dear Editor,

Thank you for the opportunity to review our manuscript. Our detailed responses to the reviewers' comments can be found in the new file titled "PLOS_One-Decision_REVIEWERS_LETTER".

Please do not hesitate to contact us if you require further information.

Sincerely,

Ass.Prof. Dr. Sonia Prado López

Attachment Submitted filename: PLOS_ONE_Decision_REVIEWERS_LETTER.pdf

# Peer review: Decision Letter 1

**DOI:** 10.1371/journal.pone.0357972.r003

**Authors:** Xiaozhe Han

21 Jul 2026

Dear Dr. Prado López,

Thank you for submitting your manuscript to PLOS ONE. After careful consideration, we feel that it has successfully addressed most of the reviewers' critiques but some experimental details does not fully meet PLOS ONE’s publication criteria as it currently stands. Therefore, we invite you to submit a revised version of the manuscript that addresses the points raised by reviewer 2.

Please submit your revised manuscript by Sep 04 2026 11:59PM. If you will need more time than this to complete your revisions, please reply to this message or contact the journal office at plosone@plos.org. When you're ready to submit your revision, log on to https://www.editorialmanager.com/pone/ and select the 'Submissions Needing Revision' folder to locate your manuscript file.

- A letter that responds to each point raised by the academic editor and reviewer(s). You should upload this letter as a separate file labeled 'Response to Reviewers'.
- A marked-up copy of your manuscript that highlights changes made to the original version. You should upload this as a separate file labeled 'Revised Manuscript with Track Changes'.
- An unmarked version of your revised paper without tracked changes. You should upload this as a separate file labeled 'Manuscript'.

If you would like to make changes to your financial disclosure, please include your updated statement in your cover letter. Guidelines for resubmitting your figure files are available below the reviewer comments at the end of this letter.

If applicable, we recommend that you deposit your laboratory protocols in protocols.io to enhance the reproducibility of your results. Protocols.io assigns your protocol its own identifier (DOI) so that it can be cited independently in the future. For instructions see: https://journals.plos.org/plosone/s/submission-guidelines#loc-laboratory-protocols. Additionally, PLOS ONE offers an option for publishing peer-reviewed Lab Protocol articles, which describe protocols hosted on protocols.io. Read more information on sharing protocols at https://plos.org/protocols?utm_medium=editorial-email&utm_source=authorletters&utm_campaign=protocols.

As the corresponding author, your ORCID iD is verified in the submission system and will appear in the published article. PLOS supports the use of ORCID, and we encourage all coauthors to register for an ORCID iD and use it as well. Please encourage your coauthors to verify their ORCID iD within the submission system before final acceptance, as unverified ORCID iDs will not appear in the published article. *Only*  the individual author can complete the verification step; PLOS staff *cannot*  verify ORCID iDs on behalf of authors.

We look forward to receiving your revised manuscript.

Kind regards,

Xiaozhe Han, D.M.D., Ph.D.

Academic Editor

PLOS One

Journal Requirements:

If the reviewer comments include a recommendation to cite specific previously published works, please review and evaluate these publications to determine whether they are relevant and should be cited. There is no requirement to cite these works unless the editor has indicated otherwise.

Please review your reference list to ensure that it is complete and correct. If you have cited papers that have been retracted, please include the rationale for doing so in the manuscript text, or remove these references and replace them with relevant current references. Any changes to the reference list should be mentioned in the rebuttal letter that accompanies your revised manuscript. If you need to cite a retracted article, indicate the article’s retracted status in the References list and also include a citation and full reference for the retraction notice.

[Note: HTML markup is below. Please do not edit.]

Reviewers' comments:

Reviewer's Responses to Questions

**Comments to the Author**

Reviewer #1: All comments have been addressed

Reviewer #2: All comments have been addressed

**********

2. Is the manuscript technically sound, and do the data support the conclusions?

Reviewer #1: Yes

Reviewer #2: Yes

**********

3. Has the statistical analysis been performed appropriately and rigorously?

Reviewer #1: Yes

Reviewer #2: Yes

**********

4. Have the authors made all data underlying the findings in their manuscript fully available?

The PLOS Data policy

Reviewer #1: Yes

Reviewer #2: Yes

**********

5. Is the manuscript presented in an intelligible fashion and written in standard English?

Reviewer #1: Yes

Reviewer #2: Yes

**********

Reviewer #1: (No Response)

Reviewer #2: I appreciate the authors' careful and detailed responses to the comments raised during the first round of review. The manuscript has been substantially improved, and the authors have made a genuine effort to address the majority of the concerns. The additions to the Introduction, Methodology, Results, and Discussion have significantly strengthened the scientific quality and clarity of the manuscript.

The revised manuscript now provides clearer descriptions of the machine learning framework, the biological relevance of necrosis, the dataset preparation, model architectures, and the statistical analyses. The inclusion of a dedicated limitations section and the expanded discussion on the biological differences between HT-29 and Hs-766T spheroids are particularly valuable improvements.

A few issues remain only partially addressed. The comparison with previous studies could be further strengthened by providing a more critical discussion of related machine learning and image-analysis approaches. In addition, clearer reporting of the exact numbers of images used for the training and testing datasets would improve transparency and reproducibility. Although the statistical analysis has been strengthened, reporting confidence intervals would provide additional support for the robustness of the findings. Finally, while the omission of a sensitivity analysis is reasonably justified, this limitation should remain clearly acknowledged in the Discussion.

Overall, I believe the manuscript is now suitable for publication after these minor revisions.

**********

what does this mean?). If published, this will include your full peer review and any attached files.

If you choose “no”, your identity will remain anonymous but your review may still be made public.

**Do you want your identity to be public for this peer review?**  For information about this choice, including consent withdrawal, please see our Privacy Policy

Reviewer #1: No

Reviewer #2: **Yes:** Elaheh Dalir Abdolahinia

**********

[NOTE: If reviewer comments were submitted as an attachment file, they will be attached to this email and accessible via the submission site. Please log into your account, locate the manuscript record, and check for the action link "View Attachments". If this link does not appear, there are no attachment files.]

To ensure your figures meet our technical requirements, please review our figure guidelines: https://journals.plos.org/plosone/s/figures

You may also use PLOS’s free figure tool, NAAS, to help you prepare publication quality figures: https://journals.plos.org/plosone/s/figures#loc-tools-for-figure-preparation.

NAAS will assess whether your figures meet our technical requirements by comparing each figure against our figure specifications.

# Author comment: Author response to Decision Letter 2

**DOI:** 10.1371/journal.pone.0357972.r004

20 Aug 2026

Reviewer’s answers

We thank the reviewers for their thoughtful feedback and constructive suggestions. We have carefully addressed each of the remaining remarks to strengthen our manuscript. Bellow, we provide point by point responses outlining the revisions made on the text:

1.The comparison with previous studies could be further strengthened by providing a more critical discussion of related machine learning and image-analysis approaches.

We appreciate this suggestion since this was only partially addressed in the previous manuscript version. To provide a more thorough and critical context, we have restructured and extended the paragraph in the Introduction surveying state of the art (SOTA) computer vision methods applied to spheroids (Page 3, lines 64-70):

“Computer Vision (CV) has proven effective in similar contexts; however, existing state of the art methods focuses primarily on studying spheroid viability (extracted as a ratio from 0 to 1)13,14, or extracting growth KPIs15 from single Brightfield images using mostly Deep Learning Methods.

While these approaches offer valuable methodological insights, they address fundamentally different task formulations. Specifically, image to scalar regression. Our framework, nonetheless, requires image to image (or image to mask) translation models. Consequently, direct benchmarking against these pre-existing models was not methodologically feasible.”

2. In addition, clearer reporting of the exact numbers of images used for the training and testing datasets would improve transparency and reproducibility.

We agree that precise reporting of dataset partitioning is essential for reproducibility. We have updated the Material and Methods section to clearly indicate when a split is needed and the split proportions. The exact number of replicates allocated to each split is provided in Table I (Page 7, lines 181-184):

“For classical Machine Learning algorithms (Logistic Regression and Decision Trees), a 70/30 train/test split was applied. The exact number of spheroid replicates corresponding to these splits is detailed in Table I. For Deep Learning architectures, a 5-fold cross-validation scheme was implemented to mitigate overfitting and ensure robust performance evaluation.”

3. Although the statistical analysis has been strengthened, reporting confidence intervals would provide additional support for the robustness of the findings.

We strongly agree with the reviewer, especially as it was reported in the discussion that the score distribution is not always gaussian, where traditional standard deviation bounds (e.g., 0.911 ± 0.118) can be misleading. Therefore, we have replaced all the standard deviation metrics with non-parametric Bootstrap Mean-based 95% confidence intervals. We believe this provides a significantly more accurate representation of our results. The updated metrics have been incorporated through Table II (pages 11-12, line 316).

4. Finally, while the omission of a sensitivity analysis is reasonably justified, this limitation should remain clearly acknowledged in the Discussion.

We appreciate this point. To ensure complete transparency, we have added an explicit statement acknowledging this limitation at (page 16, lines 411-414):

“Finally, performing standardized sensitivity analysis for image-to-image deep learning models remains complex and susceptible to interpretive bias. Instead of applying sensitivity analysis, we prioritize analysing the underlying performance distributions, directly evaluating the advantages, disadvantages, and specific failure modes for each model architecture and image modality.”

Attachment Submitted filename: Reviewers_answers_PLOSOne_20Aug26.docx

# Editor report: Decision Letter 2

**DOI:** 10.1371/journal.pone.0357972.r005

**Authors:** Xiaozhe Han

26 Aug 2026

Machine Learning-supported Identification of Necrosis in Colorectal and Pancreatic Cancer Spheroids.

PONE-D-26-05059R2

Dear Dr. Prado López,

We’re pleased to inform you that your manuscript has been judged scientifically suitable for publication and will be formally accepted for publication once it meets all outstanding technical requirements.

Within one week, you’ll receive an e-mail detailing the required amendments. When these have been addressed, you’ll receive a formal acceptance letter and your manuscript will be scheduled for publication.

An invoice will be generated when your article is formally accepted. Please note, if your institution has a publishing partnership with PLOS and your article meets the relevant criteria, all or part of your publication costs will be covered. Please make sure your user information is up-to-date by logging into Editorial Manager at Editorial Manager® and clicking the ‘Update My Information' link at the top of the page. For questions related to billing, please contact billing support.

If your institution or institutions have a press office, please notify them about your upcoming paper to help maximize its impact. If they’ll be preparing press materials, please inform our press team as soon as possible -- no later than 48 hours after receiving the formal acceptance. Your manuscript will remain under strict press embargo until 2 pm Eastern Time on the date of publication. For more information, please contact onepress@plos.org.

Kind regards,

Xiaozhe Han, D.M.D., Ph.D.

Academic Editor

PLOS One

Additional Editor Comments (optional):

Reviewers' comments:

# Editor report: Acceptance letter

**DOI:** 10.1371/journal.pone.0357972.r006

**Authors:** Xiaozhe Han

PONE-D-26-05059R2

PLOS One

Dear Dr. Prado-López,

I'm pleased to inform you that your manuscript has been deemed suitable for publication in PLOS One. Congratulations! Your manuscript is now being handed over to our production team.

At this stage, our production department will prepare your paper for publication. This includes ensuring the following:

* All references, tables, and figures are properly cited

* All relevant supporting information is included in the manuscript submission,

* There are no issues that prevent the paper from being properly typeset

You will receive further instructions from the production team, including instructions on how to review your proof when it is ready. Please keep in mind that we are working through a large volume of accepted articles, so please give us a few days to review your paper and let you know the next and final steps.

Lastly, if your institution or institutions have a press office, please let them know about your upcoming paper now to help maximize its impact. If they'll be preparing press materials, please inform our press team within the next 48 hours. Your manuscript will remain under strict press embargo until 2 pm Eastern Time on the date of publication. For more information, please contact onepress@plos.org.

You will receive an invoice from PLOS for your publication fee after your manuscript has reached the completed accept phase. If you receive an email requesting payment before acceptance or for any other service, this may be a phishing scheme. Learn how to identify phishing emails and protect your accounts at https://explore.plos.org/phishing.

If we can help with anything else, please email us at customercare@plos.org.

Thank you for submitting your work to PLOS One and supporting open access.

Kind regards,

PLOS One Editorial Office Staff

on behalf of

Dr. Xiaozhe Han

Academic Editor

PLOS One
